# The Venom of *Vipera ammodytes ammodytes*: Proteomics, Neurotoxic Effect and Neutralization by Antivenom

**DOI:** 10.3390/vetsci11120605

**Published:** 2024-11-28

**Authors:** Saša R. Ivanović, Dina Rešetar Maslov, Ivana Rubić, Vladimir Mrljak, Irena Živković, Nevena Borozan, Jelica Grujić-Milanović, Sunčica Borozan

**Affiliations:** 1 Department of Pharmacology and Toxicology, Faculty of Veterinary Medicine, University of Belgrade, Bulevar Oslobođenja 18, 11000 Belgrade, Serbia; 2 Laboratory of Proteomics, Clinic for Internal Diseases, Faculty of Veterinary Medicine, University of Zagreb, Heinzelova 55, 10000 Zagreb, Croatia; drmaslov@vef.unizg.hr (D.R.M.); irubic@vef.unizg.hr (I.R.); vmrljak@vef.hr (V.M.); 3 Institute of Virology, Vaccines and Sera “Torlak”, Vojvode Stepe 458, 11000 Belgrade, Serbia; izivkovic@torlak.rs; 4 Faculty of Medicine, University of Belgrade, Dr Subotića 1, 11000 Belgrade, Serbia; nevena.borozan@yahoo.com; 5 Department of Cardiovascular Research, Institute for Medical Research, National Institute of the Republic of Serbia, University of Belgrade, 11000 Belgrade, Serbia; jeca@imi.bg.ac.rs; 6 Department of Chemistry, Faculty of Veterinary Medicine, University of Belgrade, Bulevar Oslobođenja 18, 11000 Belgrade, Serbia; sborozan@vet.bg.ac.rs

**Keywords:** proteomics, venom, *Vipera ammodytes ammodytes*, neurotoxicity, diaphragm, Na^+^/K^+^-ATPase, antivenom

## Abstract

Deep proteomic analyses identified, in total, 159 master proteins (with 1% FDR and 2 unique peptides) from 26 protein families in the venom of *Vipera ammodytes ammodytes (Vaa).* Data are available via ProteomeXchange with the identifier PXD056495. The relative abundance of PLA2s is 11.60% of the crude venom, of which 4.35% are neurotoxic Ammodytoxins (Atxs). The neurotoxicity of the venom of *Vaa* and the neutralizing effect of the antivenom were tested on the neuromuscular preparation of the diaphragm (NPD) of rats. The activity of PLA2 in the venom of *Vaa* and its neutralization by the antivenom were determined under in vitro conditions. The *Vaa* venom leads to a progressive decrease in NPD contractions. We administered pre-incubated venom/antivenom mixtures at various ratios of 1:2, 1:10 and 1:20 (*w*/*w*) and observed the effects of these mixtures on NPD contractions. The results show that the mean effective time (ET_50_) for NPD contractions with the 1:20 mixture is highly significantly different (*p* < 0.001) from the ET_50_ for the venom and the ET_50_ for the 1:2 and 1:10 mixture ratios. We also found a highly significant (*p* < 0.001) reduction in Na^+^/K^+^-ATPase activity in the NPD under the influence of the venom. The reduction in the activity of this enzyme was reversible by the antivenom. Under in vitro conditions, we have achieved the complete neutralization of PLA2 by the antivenom. In conclusion, the antivenom abolished the venom-induced progressive decrease in NPD contractions in a concentration-dependent manner. Antivenom with approximately the same mass proportion almost completely restores Na^+^/K^+^-ATPase activity in the NPD and completely neutralizes the PLA2 activity of the venom in vitro.

## Simple Summary

In this study, we first investigated the composition of the crude venom of the most venomous European snake, *Vipera ammodytes*, using proteomic analysis. The venom of this snake contains β-neurotoxins—phospholipases A2; therefore, the neurotoxic effect was tested on neuromuscular preparations of the diaphragm of rats. We have shown that venom leads to a progressive decrease in the contractility of the diaphragm. After the administration of the antivenom, a protective effect was achieved, as the progressive decrease in diaphragm contractility was abolished. In addition, the antivenom completely neutralized the activity of phospholipases A2 under in vitro conditions. The investigation of the activity of Na^+^/K^+^-ATPase in the neuromuscular preparation of the diaphragm showed that the venom leads to a pronounced reduction in the activity of this enzyme. This reduction in enzyme activity was reversible, as the antivenom almost completely restores the activity of Na^+^/K^+^-ATPase in the neuromuscular preparation of the diaphragm.

## 1. Introduction

After the WHO classified venomous snake bites as Neglected Tropical Diseases (NTDs), there was a global scientific call to increase interest in new approaches to developing antivenoms [1,2,3].

**Figure 1:**
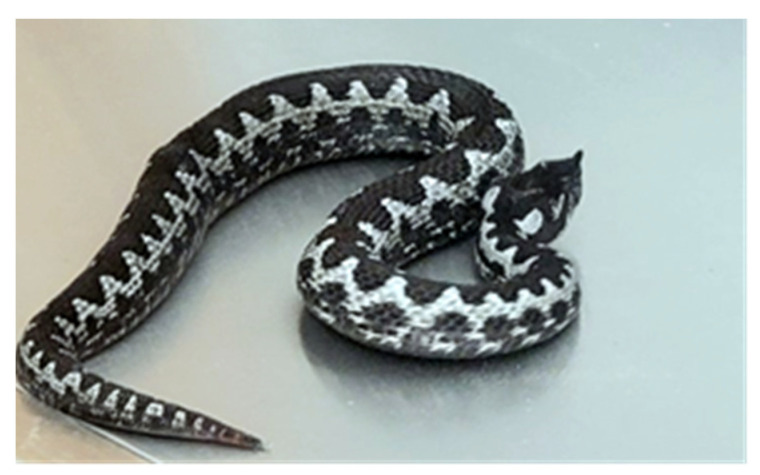
*Vipera ammodytes ammodytes*. Original photo: Institute of Virology, Vaccines and Sera “Torlak”, Belgrade, Serbia.

*Vaa* (Figure 1) is the most common venomous snake in Serbia, both in terms of numbers and distribution area, and at the same time, its venom has the highest toxicity [4].

Snake venoms have a very complex and chemically heterogeneous composition, in which the most abundant proteins and peptides play an important role in the immobilization and digestion of prey. Some of the snake venom proteins interfere with important biological processes in mammals, such as blood clotting and blood pressure regulation, or have toxic effects, including myotoxic, cardiotoxic and neurotoxic effect [5,6]. The main clinical manifestations of the neurotoxic effect of snake venom result from acute neuromuscular paralysis and include bulbar palsy with ptosis of the eyelids, hypotonia of the extremities and respiratory insufficiency as the clinically most important neurotoxic effect [7,8]. However, there are still no predictive models for the development of respiratory insufficiency that would significantly contribute to timely and appropriate decision-making in clinical therapy. The involvement of respiratory insufficiency during envenomation and the response to antivenom depend on the snake species, i.e., the mechanism of neurotoxicity of its venoms [9,10,11]. Ranawaka et al. [9] list nine potential sites of action for snake neurotoxins at the neuromuscular synapse, but there are basically two types of inhibitions of neuromuscular transmission: presynaptic (β-neurotoxins) and postsynaptic (α-neurotoxins) [12]. The dominant paralytic toxins of snake venoms are the three-finger toxins (3FTx), non-enzymatic α-neurotoxins found exclusively in the venom of Elapidae and phospholipases A2 (PLA2), enzymatic β-neurotoxins found in the venom of both Elapidae and Viperidae. The toxins 3FTx and PLA2 cause a disturbance of neurotransmission at the neuromuscular synapse by different mechanisms [13]. Due to its complexity, the neurotoxic mechanism of PLA2 is still the subject of intensive research. The initial phase of PLA2 action involves the hydrolysis of phospholipids in the neurilemma of the presynaptic motor nerve terminal [14,15]. The resulting destabilization of the lipid bilayer of the cell membrane allows for a considerable influx of Ca^2+^ ions from the extracellular space into the cell. This uncontrolled influx of Ca^2+^ further stresses the damaged cell membrane and leads to mitochondrial dysfunction, resulting in reduced ATP production [16,17,18]. There is also evidence that PLA2 from the venoms of Elapidae and Viperidae leads to the inhibition of the enzyme Na^+^/K^+^-ATPase in various cells, including erythrocytes [19], cardiomyocytes [20,21], neurons of the brain [22,23] and smooth muscle cells [24,25].

The only specific therapy for snake envenomation is the administration of an antivenom. Important facts about the production, type and use of antivenom in Europe are listed in the next part. There are eight antivenoms for bites from *Vipera* species. The Material Safety Data Sheet (MSDS) was available for seven of them (Poland, Bulgaria, Turkey, Serbia, United Kingdom, Croatia and France; no SDS could be obtained for the Russian antivenom). None of these antivenoms are licenced by the European Medicines Agency (EMA). Of the seven antivenoms with an MSDS, six are equine antivenoms and one is a ovine antivenom. The equine antivenoms are F(ab′)2 products, while the ovine antivenom is a Fab product [26]. The antivenom from Serbia is an equine F(ab′)2 product. Within the family Viperidae, six antivenoms have been produced against the venom of *V. berus* or *V. ammodytes*, one against the venoms of *V. ammodytes*, *V. aspis* and *V. berus* and one against the venoms of *V. ammodytes*, *Macrovipera lebetina* and *Montivipera xanthina*. In Europe, there is no standardized protocol for the administration of antivenom. Six manufacturers recommend intramuscular administration, while two manufacturers recommend intravenous administration of the antivenom [26,27].

Proteomic analyses have shown that the venom of *V. berus* is less complex than that of *V. ammodytes*. A particularly important finding is that the content of the PLA2 is lower in *V. berus* compared to that in *V. ammodytes*. Therefore, antivenoms developed against the venom of *V. berus* are not sufficiently effective in the treatment of severe cases of *V. ammodytes* bites [27,28]. It is obvious that the variability in the composition of the venom has a decisive influence on the design of antivenoms. In this context, proteomic analyses are of great importance as they provide fundamental knowledge about the components of the venom. On the basis of this knowledge, progress can be expected with regard to the two most important requirements for the development of antivenoms: greater efficacy and reduced side effects [29,30]. Recent literature data show that the application of modern proteomics has led to the characterization of the venom of 30% of snakes of the family Viperidae and 17% of snakes of the family Elapidae [31].

Our proteomic analysis of *Vaa* venom has identified the largest number of different proteins and protein families to date. To our knowledge, this is the first study to demonstrate the protective effect of an antivenom against the neurotoxic effect of the venom of *Vaa* using neuromuscular preparations of the diaphragm. We hypothesized that the neurotoxic effect of PLA2 involves the inhibition of Na^+^/K^+^-ATPase activity and showed that the venom of *Vaa* significantly inhibits this enzyme. Finally, a very strong positive correlation between the activity of the Na^+^/K^+^-ATPase and the increasing concentration of the antivenom was demonstrated.

## 2. Materials and Methods

### 2.1. Chemicals and Animals

All reagents were purchased from Sigma Aldrich (St. Louis, MO, USA) and Fluka (Jerusalem, Israel).

The venoms used in this study were collected by “milking” adult *Vaa* (14 males and 6 females) originating from different parts of the Republic of Serbia. The snakes were identified by herpetologists from the Institute of Virology, Vaccines and Sera “Torlak” (Belgrade, Serbia). In this institute, the snakes are kept in a serpentarium at a controlled temperature (23–26 °C) and humidity (60%). The venom obtained from them was stored at −20 °C before and after lyophilization.

The antivenom “Viekvin” produced by the Institute of Virology, Vaccines and Sera “Torlak” (Belgrade, Serbia) was obtained by the immunization of healthy mares aged 5 to 10 years with a body weight of more than 600 kg. The production process of the snake antivenom “Viekvin” was described in our previous article [32].

The preparation “Viekvin” is a solution for injection or intramuscular or intravenous administration. In total, 1 mL of the preparation neutralizes not less than 100 LD_50_ of long-nosed viper venom (*V. ammodytes*) and 50 LD_50_ of common European adder venom (*V. berus*). Marketing Authorisation number and Date: 515-01-01036-21-001, 20 September 2021, Medicines and Medical Devices Agency of Serbia (ALIMS) (https://torlak.rs/wp-content/uploads/2024/02/PIL-VIEKVIN-Eng.pdf (accessed on 1 November 2024)).

The isolated rat diaphragm model was used to investigate the neurotoxicity of *Vaa* venom (Section 2.3). Male Wistar rats weighing 200 ± 20 g were used. The rats were kept under standard laboratory conditions, with a 12 h light/dark cycle, a room temperature of 21–24 °C and *ad libitum* access to standard food and water.

The Ethics Committee of the Institute of Virology, Vaccines and Sera “Torlak”, Republic of Serbia and the Veterinary Directorate of the Ministry of Agriculture of the Republic of Serbia have approved all procedures used in this study (No. 323-07-02181/2021-05, 8 March 2021), which are in accordance with the EU Directive 2010/63/EU on the protection of animals used for scientific purposes.

### 2.2. Proteomics of the Vaa Venom

#### 2.2.1. Preparation of Protein Fractions

Lyophilised venom (1 mg) was dissolved to an appropriate concentration in 750 µL of 0.1 M triethylammonium bicarbonate (TEAB, Thermo Scientific, Rockford, IL, USA). Solubilization was enhanced by vortex mixing followed by centrifugation (14,000× *g*, 10 min, 4 °C). The resulting supernatant (fraction 0) represents the total protein solution (venom). Fraction 0 was prepared in duplicate, with the first used for proteomics and the second for the preparation of the protein fractions.

The DiffPOP (differential precipitation of proteins) method was applied as previously described [33] to generate venom protein fractions (3A, 5A, 8A, 9A and 10A). In summary, fraction 0 was sequentially mixed with acidified methanol that served as a destabilizer (1% acetic acid in 90% methanol (LC-MS Cromasolv, Honeywell, Riedel-de Haën, Charlotte, NC, USA) in ultrapure water, *v*/*v*). This process led to the precipitation of a subset of proteins after rigorous vortex mixing and subsequent centrifugation (14,000× *g*, 10 min, 4 °C). The supernatant containing the remaining proteins was then transferred to another low-protein binding tube, and the destabilizer was added again followed by centrifugation to obtain another protein pellet. This step was repeated a total of five times, with the volumes of added acidified methanol being 8, 25, 85, 130 and 425 µL. Subsequently, the collected DiffPOP protein pellets (n = 5) were washed with ice-cold acetone and centrifuged (14,000× *g* for 10 min at 4 °C). The resulting pellets were air-dried and dissolved in 0.1 M TEAB for sample preparation for proteomics.

#### 2.2.2. Sample Preparation for Proteomics

The total protein concentrations in fractions 0, 3A, 5A, 8A, 9A and 10A were determined using a Pierce BCA protein assay kit (Thermo Scientific, Rockford, IL, USA) according to the manufacturer’s protocol. For the protein digestion workflow, we started with 35 µg proteins per fraction, adjusting the final volume to 50 µL per sample by adding 0.1 M TEAB. We followed the procedure described by [34] for reduction, alkylation and trypsin digestion. Initially, we reduced the proteins using a 200 mM dithiothreitol solution at 55 °C for 60 min, followed by alkylation with a 375 mM solution at RT for 30 min in the dark. After overnight precipitation with acetone at −20 °C, the protein pellets were dissolved in 50 μL of 0.1 M TEAB following centrifugation. Trypsin Gold, a mass-spectrometry-grade trypsin powder from Promega, was prepared as a 1 mg/mL solution by adding 0.1 M TEAB. Trypsin solution was added to the protein aliquots at a trypsin-to-protein ratio of 1:35, and digestion was performed overnight at 37 °C. We then vacuum-dried peptide aliquots (10 µL) and prepared them for nano-LC–MS/MS analysis.

#### 2.2.3. Nano-Liquid Chromatography Tandem Mass Spectrometry-Based Proteomics

We performed high-resolution nano-LC–MS/MS separation and detection of peptides on the UltiMate 3000 RSLCnano system (Thermo Fisher Scientific, Germering, Germany) coupled to the Q Exactive Plus Hybrid Quadrupole-Orbitrap mass spectrometer (Thermo Fisher Scientific, Bremen, Germany). Before nano-LC–MS/MS analysis, we dissolved vacuum-dried peptides in a loading solvent solution (0.1% formic acid (*v*/*v*) (VWR International, Darmstadt, Germany) in 2% acetonitrile (*v*/*v*) (Honeywell, Charlotte, NC, USA) diluted in ultrapure water (Supelco, Bellefonte, Pennsylvania, PA, USA). Peptide trapping and desalting, nano-LC–MS/MS analysis and Top8 data-dependent acquisition (DDA) in a positive-ion mode followed our previously reported method [35]. Peptide trapping occurred for 12 min at a flow rate of 15 μL/min using a C18 PepMap100 (5 μm, 100 A, 300 μm × 5 mm) trap column and a PepMap™ RSLC C18 (50 cm × 75 μm) analytical column purchased from Thermo Fisher Scientific. Peptide separation on the analytical column utilized a linear chromatographic gradient, as described in our earlier work. During peptide separation, we used two mobile phases: mobile phase A (0.1% formic acid in water (*v*/*v*)) and mobile phase B (0.1% formic acid (*v*/*v*) in 80% acetonitrile (*v*/*v*) diluted in ultrapure water). The flow rate during peptide separation was 300 nl/min. The mass spectrometer operated in a full MS scan mode with a resolution of 70.000 and an injection time set to 120 ms. We set the AGC target to 1 × 10^6^ ± 2.0 Da and applied dynamic exclusion for 30 s. HCD fragmentation was performed using collision energy (29% and 35% NCE) with a resolution of 17.500 and an AGC target of 2 × 10^5^. Peptide precursor ions without an assigned charge state and with a charge state above +7 were not fragmented.

#### 2.2.4. Data Processing, Statistics and Bioinformatics Analysis

Raw data processing and protein identification were carried out utilizing the Proteome Discoverer software (v.2.3., Thermo Fisher Scientific, Waltham, MA, USA), employing the SEQUEST algorithm and separate database (DB) search against FASTA protein sequences for (a) *Serpentes* (snake ID number 8570) (UniProt/SwissProt release May, 2024, 361.116 sequences), (b) *Vipera* (Genus) (UniProt/SwissProt release Februry, 2024, 238562 sequences) and (c) *V. ammodytes* (UniProt/SwissProt release Februry, 2024, 90 sequences). The parameters in the Proteome Discoverer software were configured as follows: up to two missed trypsin cleavage sites were allowed, with precursor and fragment mass tolerances set at 10 ppm and 0.02 Da, respectively. The carbamidomethylation of cysteine was used as a fixed modification, while the oxidation of methionine was a dynamic modification. The false discovery rate (FDR) for peptide identification was calculated using the Percolator algorithm. Proteins were confidently identified if they had at least two unique peptides and a false discovery rate (FDR) of 1% or less.

The lists of identified master proteins (per each fraction in a total of three files) and protein groups was exported from Proteome Discoverer as a Microsoft Excel file (Excel Professional Plus 2016) and further analyzed using the PivotTable feature. The lists were specifically analyzed to identify (a) the list of master proteins/protein groups identified exclusively in fraction 0 for all databases combined, (b) the list of master proteins/protein groups identified for all fractions for each individual database separately and (c) the list of all identified protein groups for all fractions using different FASTA databases.

A Venn diagram was generated using the Venn diagram tool (https://bioinformatics.psb.ugent.be/webtools/Venn/ (accessed on 1 November 2024)) by inputting protein accession numbers. This step aimed to (a) assess the protein distribution for fraction 0 using different FASTA databases, (b) evaluate the protein distribution for all fractions using *Serpentes* DB, (c) assess protein distribution for all fractions using *Vipera* DB, (d) evaluate the protein distribution for all fractions using *V. ammodytes* DB and (e) analyze the protein distribution (master proteins, two unique peptides, 1% FDR) for all fractions using different FASTA databases.

For the latter identified master proteins, if available, the alternative names, cellular component, molecular function, biological process and function in detail were copied from Uniprot/SwissProt (15–28 May 2024).

The mass spectrometry proteomics data have been deposited to the ProteomeXchange Consortium via the PRIDE partner repository with the dataset identifier PXD056495.

### 2.3. Examination of the Contractility of the Neuromuscular Preparations of the Diaphragm (NPD)

The Wistar rats were sacrificed under general anesthesia (ketamine 100 mg/kg + xylazine 10 mg/kg, intraperitoneal) by the dislocation of the cervical spine. Immediately after the sacrifice, the complete diaphragm of the rats was removed together with the bony base consisting of the ribs and sternum. The diaphragm hemispheres were cut into strips, with the cuts running parallel to the direction of the muscle fibers. The neuromuscular preparation of the diaphragm (NPD) was placed in a 20 mL organ bath containing Tyrode’s solution (composition in mM: NaCl 139.9; KCl 2.7; CaCl_2_ 1.8; MgCl_2_ 1.04; NaHCO_3_ 11.9; NaH_2_PO_4_ 0.4 and glucose 5.5, pH 7.4) at 37 °C. Tyrode’s solution was continuously aerated with a mixture of oxygen (95%) and carbon dioxide (5%). In the organ bath, the NPD is positioned in the central space between two parallel platinum electrodes connected to a BioSmart 150 stimulator (ElUnit, Serbia), which enables electrical field stimulation (EFS). The contractions were recorded in real time using the eLAB44 software (ElUnit, Serbia). The NPD is exposed to an initial tension of 1 g and waits at least 15 min until it has reached a constant basal tone and a stable amplitude of contractions. The EFS was performed with tetanic pulses, a series of “packages” with 5 stimulations every 30 s. The pauses between the “packages” were 5, 15 or 30 min, depending on the work protocol. The following parameters were used for indirect EFS: 35 Hz, 20 µs, 2 s and 15 V; and the following were used for direct EFS: 100 Hz, 500 µs, 2 s and 50 V. The maximum contraction values reached were measured and the possible occurrence of tetanic fade after the administration of the venom was monitored. The non-depolarizing neuromuscular blocker pancuronium (in phosphate buffered saline—PBS) was used to prove that the indirect EFS technique induces muscle contractions only via the mediation of the neuromuscular synapse. Pancuronium was administered at a concentration of 1 μM during contractions of the NPD without the presence of the venom and at a concentration of 3 μM during contractions under the influence of the venom.

In the initial phase of the study, different concentrations of the crude venom were administered and the contractility of the NPD was monitored as a function of time. For these tests, a stock solution of the crude venom was used at a concentration of 20 mg/mL PBS, from which a series of dilutions were prepared (from 0.10 to 50 µg/mL). In the final tests, a concentration of 35 µg/mL of crude venom was used as the appropriate concentration as it resulted in an almost complete inhibition of NPD contractility (over 90% inhibition compared to the control). The protective effect of antivenom on the contractility of NPD was investigated using venom/antivenom mixtures at different mass ratios (*w*/*w*): 1:2, 1:10 and 1:20. The mixtures were pre-incubated for 30 min at a constant temperature of 37 °C [36]. The protein concentration of the antivenom used for these tests was 100 mg/mL. In order to compare the protective effects of different venom/antivenom mixtures (1:2; 1:10; 1:20), the mean effective time (ET_50_) was used. Logarithmic functions of time (log_10_, minutes), the normalization of effects (% of control NPD contractions) and non-linear regression were used to calculate the ET_50_ value. After the completion of the NPD contractility tests, AChE and Na^+^/K^+^-ATPase activity was determined in the same preparations.

### 2.4. Determination of the Activity of Acetylcholinesterase (AChE) and Sodium/Potassium ATPase (Na^+^/K^+^-ATPase) in the NPD

The diaphragmatic tissue samples were homogenized in a buffer containing 50 mM Tris HCl pH 7.4; 20 mM EDTA, 1 M NaCl and 1% Triton-X100 according to the method of Krummer et al. [37]. An Ultra Turrax homogenizer (Janke and Kunkel IKA Works GmbH & Co. KG Staufen, Staufen/Germany) was used for homogenization. The ratio of tissue-to-buffer was 1:5 (*w*/*v*), and homogenization was performed on ice. The homogenate was centrifuged at 20,817× *g* at +4 °C. The supernatant was then poured off, stored at −20 °C and used for further analyses.

AChE activity in the diaphragm was determined using acetylthiocholine iodide as the substrate according to Ellman et al. [38]. The reaction was monitored spectrophotometrically over a period of 5 min at 412 nm. The results are expressed in units per milligram protein of the diaphragm (U/mg P). The protein concentration was determined according to the method of Lowry et al. [39], using bovine serum albumin (BSA) as a standard.

The determination of Na^+^/K^+^-ATPase activity in the diaphragm was carried out according to the method of Pari and Murugavel [40]. The supernatant of the diaphragm was added to a reaction mixture containing 50 mM Tris HCl, 5 mM MgCl_2_, 100 mM NaCl, 20 mM KCl and pH 7.5 and the mixture was then incubated at 37 °C in the presence of 10 mM ATP. The reaction was stopped by adding cold trichloroacetic acid (TCA) solution. After protein precipitation and centrifugation at 10,621× *g*, the concentration of released inorganic phosphorus in the supernatant was determined by incubating the mixture with a solution of (NH_4_)_6_MO_7_O_24_ and vitamin C. The absorbance was then measured at 620 nm. The enzyme activity was expressed by the concentration of inorganic phosphorus in U/mg P, and a 3 mM solution of primary potassium phosphate was used as a standard.

### 2.5. Activity of Venom Phospholipase A2 (PLA2) and Neutralization by Antivenom

The activity of PLA2 of the venom of *Vaa* was determined according to the modified methods of Tan and Tan [41]. A fresh solution of the substrate was prepared by mixing egg yolk (380 g) with 18.1 mM sodium deoxycholate and 8 mM CaCl_2_ at a ratio of 1:1:1 (*v*/*v*/*v*) overnight at room temperature. The pH was adjusted to 8.1 with 0.15 M NaOH. The venom solution was added to a substrate at a concentration range of 0.05 to 1 mg/mL, and the pH change was observed for 60 s. All measurements were carried out in triplicate (n = 3). To test the neutralization of PLA2 by antivenom, a 1 mg/mL solution of the venom was used. Antivenom concentrations from 5 to 50 mg/mL were tested. The test was performed after the incubation of the venom/antivenom mixture at 37 °C for 30 min. The results are expressed as pH change per unit time (ΔpH/min).

### 2.6. Statistical Analysis

The data obtained were processed using GraphPad Prism 8.00 statistical software (GraphPad Software Inc., San Diego, CA, USA). The statistical analysis of the results on the neurotoxicity of the venom was performed using a one-way ANOVA followed by Tukey’s Multiple Comparison Test. Values *p* < 0.05 were considered significant. All experimental results are expressed as the mean ± SD. Correlation was tested using Pearson’s correlation analysis (r).

## 3. Results

### 3.1. Proteomics of the Vaa Venom

**Figure 2:**
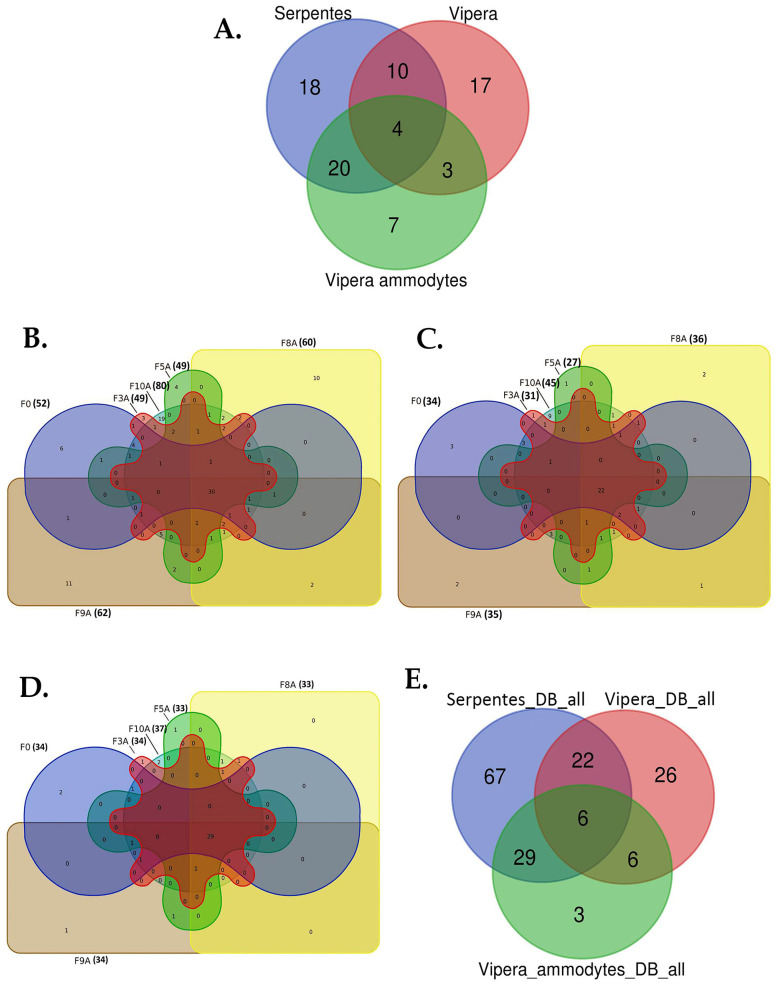
(**A**) Distribution of identified proteins for fraction 0 when different protein FASTA databases were used in the analysis; (**B**) Distribution of identified proteins for all fractions (0, 3A, 5A, 8A, 9A and 10A) when *Serpentes* protein FASTA databases (DB) were used in the analysis; (**C**) Distribution of identified proteins for all fractions (0, 3A, 5A, 8A, 9A and 10A) when *Vipera* protein FASTA databases (DB) were used in the analysis; (**D**) Distribution of identified proteins for all fractions (0, 3A, 5A, 8A, 9A and 10A) when *V. ammodytes* protein FASTA databases (DB) were used in the analysis; (**E**) Distribution of identified proteins for all fractions (0, 3A, 5A, 8A, 9A and 10A) when different protein FASTA databases were used in the analysis. Next to each fraction, the number of proteins identified in this fraction for all three databases used is given in brackets.

The numbers of identified master proteins (FDR < 1% and 2 unique peptides) are presented as Venn diagrams in Figure 2. The total ion chromatograms (TIC) for the complete venom, fraction 0 and for the DiffPOP protein fractions 3A, 5A, 8A, 9A, 10A are shown in Figure S1.

In total, 79 master proteins were identified (with 1% FDR and two unique peptides) in the complete *Vaa* venom (fraction 0, Figure 2A). The highest number of identified master proteins was reported for the proteomics approach which included the application of *Serpentes* DB (52 proteins), followed by *Vipera ammodytes* and *Vipera* DB (both 34 proteins, Figure 2A). Proteins identified by a comparison to *Serpentes* DB showed an owerlap with the other two lists of identified proteins, while a higher overlap can be observed with *Vipera ammodytes* DB (20 proteins, Figure 2A). The lists of identified proteins presented in Figure 2A, including the identification number, the description of the protein (name, organism in which it occurs), the molecular mass and the isoelectric point (pI), can be found in Supplement S1 (Table S1). The number of master proteins in all analyzed *Vaa* fractions (3A, 5A, 8A, 9A and 10A, including complete *Vaa* venom, fraction 0) is shown in Figure 2B–D, depending on which protein FASTA databases (DB) were used for protein identification. The total number of identified (1% FDR and 2 unique peptides) venom proteins in all fractions varied from 124 when *Serpentes* DB (Figure 2B) was used to 60 when *Vipera* DB (Figure 2C) was used and to 44 when *Vipera ammodytes* DB (Figure 2D) was used. A detailed list of identified master proteins presented in Figure 2B–D can be found in Supplement S2 (Tables S2–S4). The number of identified master proteins (2 unique peptides, 1% FDR) in all *Vaa* fractions (0, 3A, 5A, 8A, 9A and 10A) when different protein FASTA databases were used in the analysis is presented in Figure 2E. The Veen diagram shows the highest number of master proteins identified when Serpentes DB was applied in the analysis (in total, 124 proteins), followed by Vipera DB with 60 proteins and Vipera ammodytes DB with 44 proteins (Figure 2E). A higher degree of overlaps can be observed between identified lists of proteins for Serpentes DB with both DBs, Vipera and Vipera ammodytes (Figure 2E).

The list of corresponding protein groups identified in all fractions (including complete venom) for individual databases is provided in Supplement S3 (Tables S5–S8). The numbers of identified protein groups followed the trends presented for identified master proteins. Specifically, Serpentes DB provided 52, and Vipera and Vipera ammodytes DB both provided 34 identified protein groups for complete venom (fraction 0) (Table S5). Serpentes DB provided 118, Vipera DB provided 70 and Vipera ammodytes provided 44 identified protein groups for all analyzed fractions, including total venom (Table S9).

**Figure 3:**
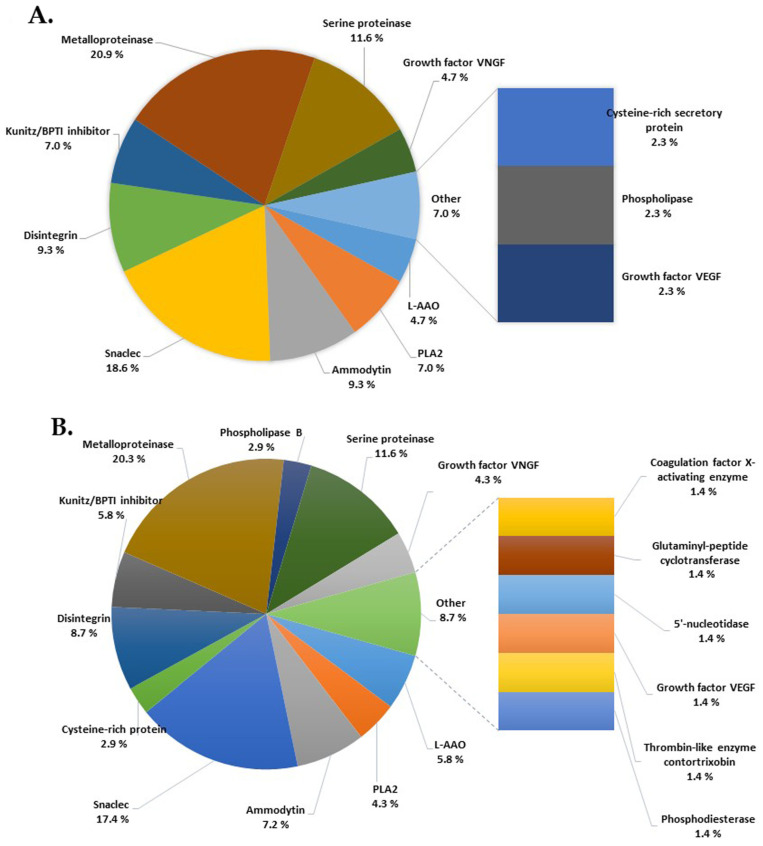
Relative distribution of protein groups (%) in the *Vaa* venom determined by nano-liquid chromatography–tandem mass spectrometry-based proteomics: (**A**) DB *V. ammodytes*; (**B**) DB *Vipera*.

The total number of master proteins identified (with 1% FDR and two unique peptides, combined for all 3 FASTA DB) in the Vaa venom in our study is 159 (Figure 2E), originating from 26 protein families (Supplement S5, Table S10). The proteins of the Vaa venom identified in our study belong to four families of enzymatic proteins: (1) Snake venom serine proteases (SvSPs), (2) L-amino acid oxidases (LAAOs), (3) Snake venom metalloproteinases (SvMPs), (4) Secretory phospholipases A2 (sPLA2s) and (5) non-enzymatic families: (1) Cysteine-rich secretory proteins (CRISPs), (2) Snake C-type lectin-like proteins (Snaclecs), (3) Venom nerve growth factors (VNGFs), (4) Vascular endothelial growth factors (VEGFs) and (5) Kunitz-type serine protease inhibitors (SPIs). Regarding the quantitative representation of the enzymes, SvMPs, sPLA2s, LAAOs and SvSPs dominate in the Vaa venom with a proportion of 53.5%. The relative abundance (%) of protein groups in the Vaa venom is shown in Figure 3.

The bioinformatic analysis identified alternative names, cellular components, molecular functions, biological processes and functions for the main proteins detected (Supplement S5, Table S10).

The proteomic analysis in our study revealed the presence of 22 isoforms of serine proteases (or serine endopeptidases—SvSPs) with molecular masses ranging from 25.1 to 38.0 kDa and pI values ranging from 5.31 to 9.19 (Supplement S2, Table S2). The relative abundance of SvSP in the venom is 11.60%.

The phospholipases (PL) of *Vaa* venom belong to group A and subgroup 2 (PLA2). Proteomic analyses have shown that the venom of *Vaa* contains several different PLA2, of which the neurotoxic Ammodytoxins (Atxs) are present in three different isoforms: AtxA, AtxB and AtxC. The venom of *Vaa* contains all three isoforms of this enzyme, which have the same molecular mass but different pI values and relative abundances in the composition of the venom (Table 1). Ammodytin L (AtnL) is a structural analog of AtxA and is enzymatically inactive but shows myotoxic and cardiotoxic effects. In addition, *Vaa* venom also contains two enzymatically active but non-toxic Ammodytins I (AtnsI): AtnI1 and AtnI2 (Table 1). The relative abundance of PLA2s in *Vaa* venom is 11.60%, of which 4.35% are neurotoxic Ammodytoxins (A, B, C), while the remaining 7.25% are Ammodytins (Atns), which have no neurotoxic effect.

*Vaa* venom is rich in metalloproteinases (SvMPs), which account for 20.30% of venom protein groups (Figure 3B). Based on their structure, snake venom metalloproteinases are divided into three classes: P-I, P-II and P-III. We have detected the presence of 53 isoforms of metalloproteinases with Uniport DB *Serpentes* (Supplement S2, Table S2). The molecular masses of these proteins range from 13.9 to 70.5 kDa, with pI values between 4.98 and 7.75. Of these 53 isoforms, 3 isoforms belong to PII MPII (Mw 53.0–53.4 kDa, pI 5.38–5.5), 4 belong to PIII MPIII (Mw 68.3–69.2 kDa, pI 5.30–6.25), 11 belong to the Zn^2+^-metalloproteinases disintegrin-like protein H4 subunit A (Mw 46.8–69.1 kDa, pI 4.88–6.38), 1 isoform is a Zn^2+^-metalloproteinase disintegrin-like ammodytagin (Mw 19.9 kDa, pI 5.15) and 34 isoforms are metalloproteinases (SvMP) (13.9–70.5 kDa, pI 4.98–7.75). Zn^2+^-dependent proteins (Zn-finger proteins) are also present in the *Vaa* venom. These are proteins with different molecular masses between 59.4 and 78.5 kDa and a pI value between 6.67 and 9.57. We have shown that there are 15 isoforms of the enzyme L-amino acid oxidase (LAAO) in *Vaa* venom: 1 isoform with a low molecular weight (Mw 10.3 kDa; pI 5.21), 2 isoforms with Mw 35.0–46.3 kDa and pI 7.72–8.76 and 12 isoforms with higher molecular weights (Mw 56.5–58.6 kDa; pI 6.49–8.54). The relative abundance of LAAO in the venom is 5.80%. Glutaminyl-peptide cyclotransferase (GPAT) is a protein with a molecular weight of 42.2 kDa and a pI value of 8.1. This enzyme is present in small amounts in *Vaa* venom, with a relative abundance of 1.40%.

Low-abundance enzymes in *Vaa* venom include thrombin-like enzymes (TLEs), which were identified with a relative abundance of 1.40%. Three isoforms were detected: two acidic (2.9 kDa; pI 4.28 and 25.4 kDa; pI 5.12) and one basic (27 kDa; pI 8.34). These are serine proteases that mimic thrombin activity and are found in the venoms of many snakes, particularly those of the Viperidae and Crotalidae families. Other enzymes with a low abundance include 5′-nucleotidase (Mw 45 kDa; pI 6.9) with a relative abundance of 1.40%, two isoforms of phosphodiesterase (PD) (Mw 91.7–96.1 kDa; pI 7.25–8.07) with a relative abundance of 1.40%, β-fibrinogenase (Mw 28.3 kDa; pI 7.34), coagulation factor-X activating enzyme heavy chain (Mw 68.7 kDa; pI 6.15 and 69.6 kDa; pI 6.9) with a relative abundance of 1.40% and phospholipase B (Mw 64.3; pI 8.5) with a relative abundance of 2.90% (Figure 3B).

Among the non-enzymatic components of *Vaa* venom, we identified Snake C-type lectin-like proteins (Snaclecs) as the most abundant non-enzymatic proteins with a prevalence of 17.40%. Proteomic analysis revealed 24 isoforms of this protein, of which 14 belong to the C-type lectins (Mw 11.8–18.7 kDa; pI 5.59–8.12) and 10 belong to the Snaclecs (Mw 12.1–18.1 kDa; pI 4.68–8.19). Disintegrins (Dis) are another family of non-enzymatic proteins in *Vaa* venom. They are common components of the venoms of Viperidae and act as integrin antagonists. Their relative abundance in *Vaa* venom is 8.70%, and we have identified 13 isoforms of this protein: 1 isoform (36.8 kDa; pI 5.03), 5 isoforms (11.5–14.0 kDa; pI 6.87–8.56) and 7 isoforms with very-small-molecular-weight proteins (7.0–7.7 kDa; pI 5.2–8.19). Cysteine-rich secretory proteins (CRISP) have also been identified. These are low-molecular-weight proteins, of which four isoforms (24.7–26.6 kDa; pI 5.96–7.46) with a relative abundance of 2.90% appear in the *Vaa* venom. Venom nerve growth factor (VNGF) (Mw 27.3 kDa; pI 8.29) was identified with an abundance of 4.30%. In addition, vascular endothelial growth factor (VEGF) (Mw 16.2 kDa; pI 5.3) and the two isoforms of VEGF toxin vammin (Mw 22.3–22.5 kDa; pI 7.83–7.96) were found with a relative abundance of 1.40% in the *Vaa* venom.

The following peptides were identified in *Vaa* venom: serine protease inhibitors—Kunitz/SPi (Mw 10.3 kDa; pI 8.7) and four isoforms of Kunitz/BPTI (Mw 9.8–10.6 kDa; pI 8.27–8.92) with a relative abundance of 5.90%; The cystatin belong to a family of cysteine protease inhibitors (Mw 12.7 kDa; pI 7.81); metalloproteinase inhibitors (MPi-3) (Mw 22.9 kDa; pI 9.06), phospholipase A2 inhibitor (Mw 22.2 kDa; pI 6.42) and endogenous tripeptide (MPi-5) (Mw 15 kDa; pI 8.06). The identified trace components include natriuretic peptide (Mw 2–3 kDa).

The proteins present in *Vaa* venom include glutathione peroxidase, lipase, deoxyribonuclease, aminopeptidase, endonuclease, amine oxidase, hyaluronidase, thioredoxin, transferrin, serum albumin-like protein and other proteins (Supplement S2, Table S2).

### 3.2. The Effects of Vaa Venom on the NPD Contractility and Protective Effect of Antivenom

**Figure 4:**
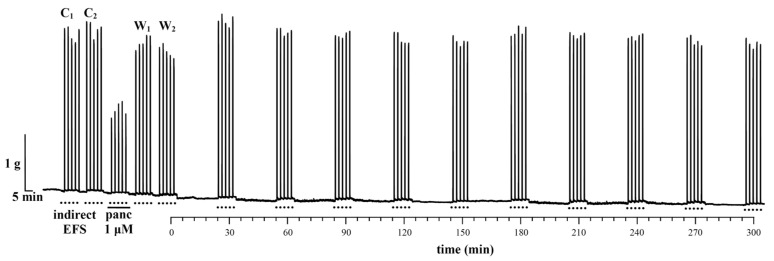
Representative recording of contractions of a neuromuscular preparation of the diaphragm (NPD) induced by indirect EFS (·····) in the absence of venom. C_1_ and C_2_—control contractions; panc 1 μM—contractions under the influence of 1 μM pancuronium; W_1_ and W_2_—contractions after the washout of pancuronium; 10 “packages” of contractions in the function of time.

In order to validate the diaphragm contractility testing methodology, a series of control NPD contractions were first performed as a function of time (in minutes). Figure 4 shows a representative recording of NPD contractions induced by EFS in the absence of venom.

The mean value of NPD contractions in the first two control “packages” (C_1_ and C_2_) is 2.88 ± 0.13 g. After the administration of 1 μM pancuronium, the contractions of the NPD decreased to 51.20% of the mean value of the contractions of the control “packages” C_1_ and C_2_. The neuromuscular blockade by pancuronium was reversible, and after its washout from the organ bath, the contractions normalized and reached the following values: the W_1_ “package” 93.02% and the W_2_ “package” 87.25% of the control contractions C_1_ and C_2_. The first 5 “packages” (C_1_, C_2_, pancuronium 1 μM, W_1_, W_2_) confirm that NPD contractions are induced by indirect EFS. Over the next 10 “packages” in a 300 min period, contractions were stable as a function of time, and 5 contractions within each “package” were uniform in the amplitude of contractions, i.e., standard deviations (±SD).

**Figure 5:**
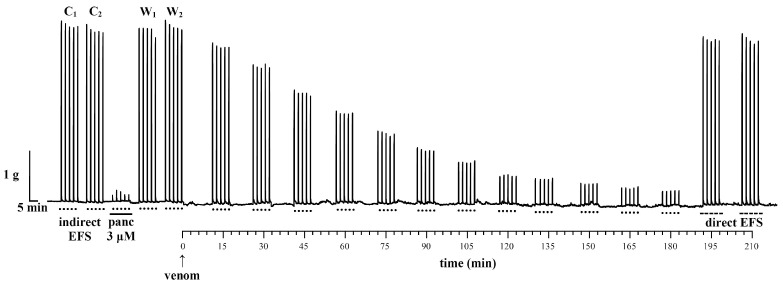
Representative recording of contractions of a neuromuscular preparation of the diaphragm (NPD) induced by indirect EFS (**·····**) and direct EFS (**-----**) under the influence of venom. C_1_ and C_2_—control contractions; panc 3 μM—contractions under the influence of 3 μM pancuronium; W_1_ and W_2_—contractions after the washout of pancuronium; 12 “packages” of contractions induced by indirect EFS; 2 “packages” of contractions induced by direct EFS.

In the next part, a series of NPD contractions were performed under the influence of the venom as a function of time. A representative recording of EFS-induced NPD contractions under the influence of the venom is shown in Figure 5.

The mean value of the NPD contractions in the first two control “packages” of contractions (C_1_ and C_2_) is 3.42 ± 0.08 g. Pancuronium at a concentration three times higher than in the previous series (3 μM) led to an almost complete blockade of NPD contractions (5.62% of control “packages” C_1_ and C_2_). The effect of the neuromuscular blockade of pancuronium was also reversible, because after the pancuronium had been washed out of the organ bath, the contractions of the “packages” W_1_ and W_2_ amounted to 97.63% and 101.14% of the control contractions, respectively. The next twelve “packages” show NPD contractions induced by indirect EFS. Under the influence of the venom, the “packages” of NPD contractions differed from each other with high statistical significance (*p* < 0.001) up to the 120th minute of exsposition. From the 120th to 180th minute, each subsequent NPD “package” of contractions did not differ statistically from the previous one (*p* > 0.05). However, a statistically significant difference was observed between the 120th and 180th minute (*p* < 0.001) and between the 135th and 180th minute (*p* < 0.01). At the end of this series, 2 “packages” of NPD contractions were induced with direct EFS parameters. The mean values of the contractions are similar to those of the control and are 95.82% (“package” at 195 min) and 94.29% (“package” at 210 min) with respect to the control “packages” C_1_ and C_2_.

Table 2 gives an overview of the percentage decrease in diaphragm contractility over time under the influence of the venom and all three tested venom/antivenom mixtures (1:2; 1:10; 1:20).

In the following three series of tests, the protective effect of the antivenom was evaluated with regard to the prevention of the progressive decline in diaphragmatic contractility under the effect of the venom. In this and the next two series of NPD contractions, we used a slightly modified protocol. To objectively evaluate the protective effect of the antivenom, we tested the stability of the NPD contractions prior to the administration of the venom/antivenom mixtures with five control “packages” (C_1_–C_5_) for 60 min.

Figure S2 shows a representative recording of EFS-induced NPD contractions under the influence of the mixture of venom/antivenom at a ratio of 1:2. The mean value of the NPD contractions in the first five control “packages” (C_1_–C_5_) is 2.14 ± 0.08 g. The next twelve “packages” show NPD contractions induced by indirect EFS. Under the influence of the venom/antivenom mixture at a ratio of 1:2, the “package” of NPD contractions after 15 min of exposure was not statistically significantly different (*p* > 0.05) from the control contractions. From the 15th to the 75th minute, each subsequent “package” of NPD contractions was statistically significantly different from the previous one (*p* < 0.01), while from the 75th to the 180th minute, each 30 min decrease in NPD contractions reached statistical significance (*p* < 0.01). The mean values of the “package” contractions (%) compared to the control contractions are listed in Table 2. The last two “packages” of NPD contractions were induced by direct EFS, and their mean values were 127.19% (“package” at 195 min) and 123.88% (“package” at 210 min) compared to the mean value of the five control “packages”.

In the penultimate test series, the mixture of venom/antivenom was used at a ratio of 1:10 (Figure S3). The mean value of the NPD contractions in the first five control “packages” (C_1_–C_5_) is 5.15 ± 0.25 g. The next twelve “packages” show NPD contractions induced by indirect EFS. Under the influence of the venom/antivenom mixture at a ratio of 1:10, the “package” of NPD contractions after 15 min of exposure was not statistically significantly different (*p* > 0.05) from the control contractions. From the 15th to the 45th minute, each subsequent “package” of NPD contractions was statistically significantly different from the previous one (*p* < 0.001). From the 45th to 105th minute, each subsequent “package” of NPD contractions did not differ statistically from the previous one (*p* > 0.05), while a statistically significant difference was observed between the 105th and 135th minute (*p* < 0.05). From the 135th to the 180th minute, each subsequent “package” of NPD contractions was statistically significantly different from the previous one (*p* < 0.01). The mean values of the “package” contractions (%) compared to the control contractions are listed in Table 2. The last two “packages” of NPD contractions were induced by direct EFS, and their mean values were 122.30% (“package” at 195 min) and 124.90% (“package” at 210 min) compared to the mean value of the five control “packages”.

In the final phase of the study, the mixture of venom/antivenom was used at a ratio of 1:20 (Figure S4). The mean value of the NPD contractions in the first five control “packages” (C_1_–C_5_) is 4.02 ± 0.11 g. Since we found in this test series that the contractions of the NPD under the influence of the venom/antivenom mixture at a mass ratio of 1:20 did not fall below 50% of the control values even after twelve “packages” of stimulation, we applied fifteen “packages” of stimulation in the last series. Under the influence of the venom/antivenom mixture, the “packages” of the NPD contractions did not differ statistically significantly (*p* > 0.05) from the contractions of the control up to the 60th minute of exposition. From the 15th to the 90th minute, the “packages” of NPD contractions did not differ statistically significantly from each other (*p* > 0.05). From the 90th to the 225th minute, each subsequent “packages” of NPD contractions was not statistically significantly different from the previous one (*p* > 0.05). The first statistically significant difference was found between the “packages” at the 90th and 195th minutes (*p* < 0.01), while the statistical significance between the 90th and 225th minutes was *p* < 0.001. The mean values of the “package” contractions (%) compared to the control contractions are listed in Table 2. It can be observed that even after 15 “packages” for 225 min, the mean value of the NPD contractions was above 50% of the control contractions (56.90% compared to the control contractions). As in the previous test series, two “packages” of contractions were induced by direct EFS at the end of this last series. Their mean values of NPD contractions are higher than those of the control and amount to 114.41% (“package” at 240 min) and 111.33% (“package” at 255 min) compared to the five “packages” of the control.

**Figure 6:**
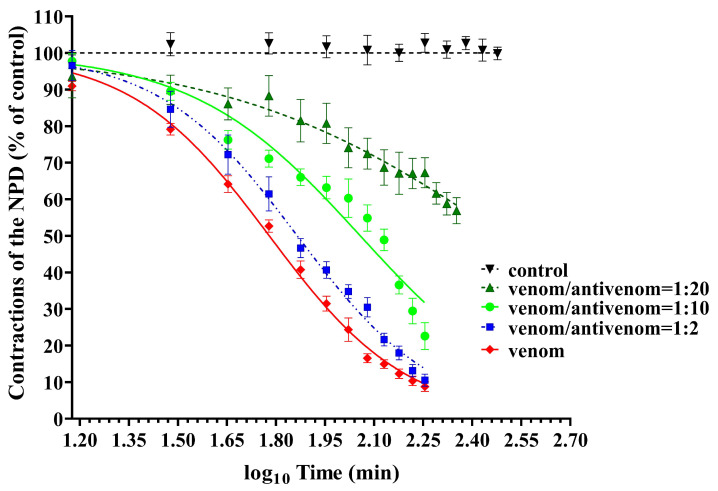
Sigmoidal curves of the reduction in contractions of the neuromuscular preparation of the diaphragm (NPD) in a logarithmic function of time under the influence of venom and venom/antivenom mixtures at ratios of 1:2; 1:10 and 1:20 (*w*/*w*).

Logarithmic functions of time (log_10_, minutes) and normalization of effects (% of control NPD contractions) were used to generate sigmoidal curves of reduction in NPD contractions under the influence of venom and venom/antivenom mixtures at different mass ratios (1:2; 1:10; 1:20) (Figure 6).

Non-linear regression was used to calculate the mean effective time (ET_50_) for which NPD contractions are reduced to 50% of control values after the administration of venom and venom/antivenom mixtures at different mass ratios (Table 3).

**Figure 7:**
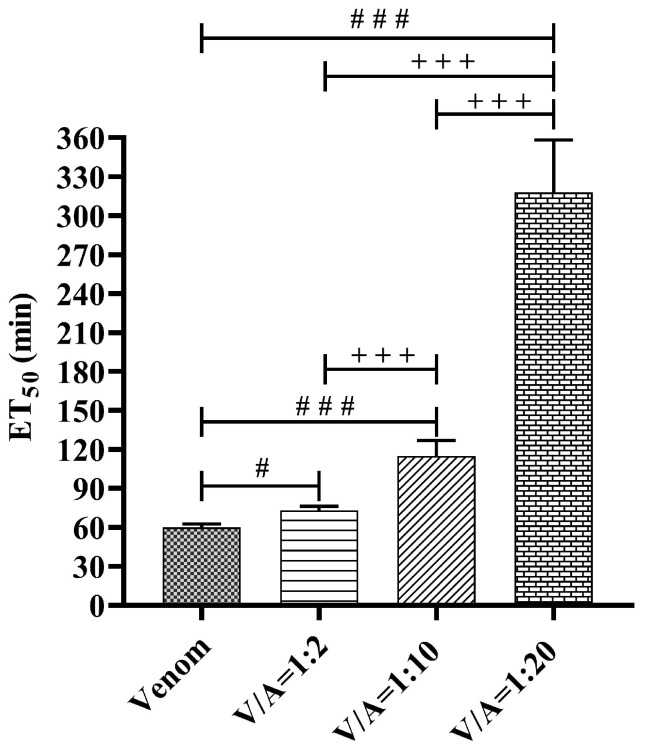
Comparison of ET_50_ (minutes) after the administration of venom and a venom/antivenom mixture at the ratios of 1:2; 1:10 and 1:20 (*w*/*w*) (mean ± SD, ^#^ *p* < 0.05, ^###^ *p* < 0.001 vs. venom; ^+++^ *p* < 0.001 between different mass ratios of venom/antivenom).

A statistically significant difference was demonstrated between the ET_50_ for the venom and the venom/antivenom mixtures at the ratios of 1:2, 1:10 and 1:20 (*w*/*w*) at the significance level of *p* < 0.05, *p* < 0.001 and *p* < 0.001, respectively. There is also a highly significant difference (*p* < 0.001) between the ED_50_ for different mass ratios of venom/antivenom (Figure 7).

**Figure 8:**
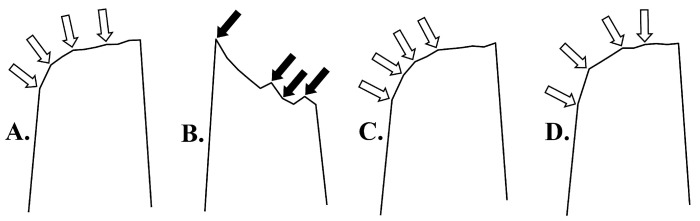
Representative recording of contraction peaks of the neuromuscular preparations of the diaphragm (NPD) induced by indirect EFS: (**A**) Control contractions; (**B**) Contractions under the influence of pancuronium *(tetanic fade)*; (**C**) Contractions under the influence of venom; (**D**) Contractions under the influence of a mixture of venom/antivenom at a ratio of 1:20 (*w*/*w*) (white arrows show a facilitated release of neurotransmitters; black arrows show a reduced release of neurotransmitters—*tetanic fade*).

As part of the investigation of NPD contractions, we examined the possible occurrence of a tetanic fade after the administration of venom and a 1:20 (*w*/*w*) venom/antivenom mixture. The magnified peaks of the NPD contractions are shown in Figure 8.

In contrast to pancuronium, which leads to a tetanic fade (Figure 8B, black arrows), the occurrence of a tetanic fade was not recorded in NPD contractions under the influence of venom (Figure 8C, white arrows) and the mixture of venom/antivenom at a ratio of 1:20 (*w*/*w*) (Figure 8D, white arrows).

### 3.3. The Effects of Vaa Venom on the Activity of AChE and Na^+^/K^+^-ATPase in the NPD

**Figure 9:**
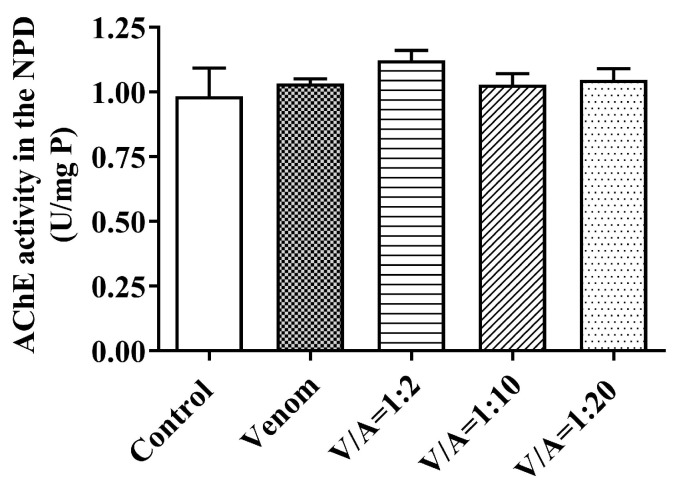
AChE activity (U/mg P) in the neuromuscular preparations of the diaphragm (NPD) without the presence of venom (control), under the influence of venom and for the mixture of venom/antivenom at a ratio of 1:2, 1:10 and 1:20 (*w*/*w*) (mean ± SD, *p* > 0.05).

No statistically significant difference (*p* > 0.05) was found in AChE enzyme activity in NPD under the influence of venom and venom/antivenom mixtures at all mass ratios tested compared to the control group (Figure 9).

**Figure 10:**
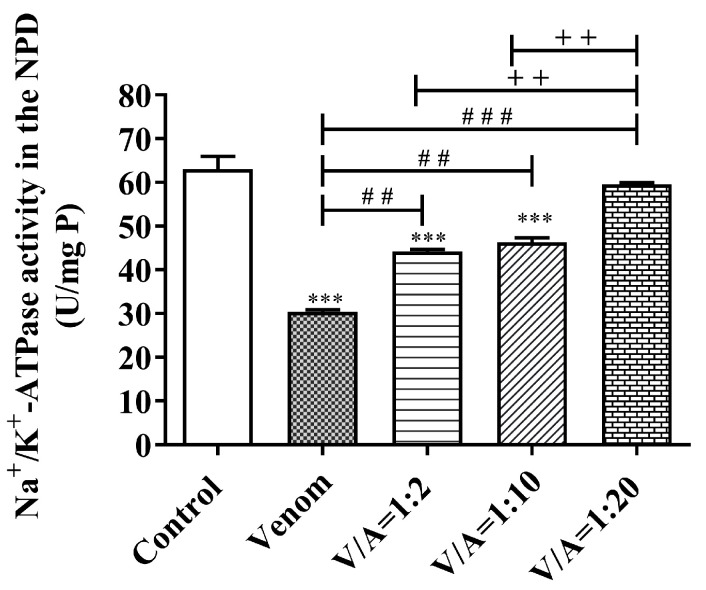
Na^+^/K^+^-ATPase activity (U/mg P) in the neuromuscular preparations of the diaphragm (NPD) without the presence of venom (control), under the influence of venom and under the influence of a mixture of venom and antivenom at the ratios of 1:2; 1:10 and 1:20 (*w*/*w*) (mean ± SD, *** *p* < 0.001 vs. control; ^##^ *p* < 0.01, ^###^ *p* < 0.001 vs. venom; ^++^ *p*<0.01 between different mass ratios of venom/antivenom).

The differences in Na^+^/K^+^-ATPase activity in all NPD samples analyzed are shown in Figure 10.

The results show that *Vaa* venom inhibits the activity of Na^+^/K^+^-ATPase highly significantly (*p* < 0.001) compared to the control (51.79% inhibition). With an increase in the mass fraction of the antivenom, the enzymatic activity of the Na^+^/K^+^-ATPase increases in comparison to the activity under the influence of the venom. The 1:2 and 1:10 venom/antivenom mixtures statistically significantly (*p* < 0.01) restore the activity of Na^+^/K^+^-ATPase compared to the effect of the venom. However, the enzyme activity remains highly significantly (*p* < 0.001) lower than the control value and after the application of the 1:10 mixture. After the application of the mixture at a ratio of 1:20, there is no longer a statistically significant difference in the activity of the Na^+^/K^+^-ATPase compared to the control (*p* > 0.05). We tested the correlation between the activity of Na^+^/K^+^-ATPase in the NPD after applying different ratios of venom/antivenom: 1:2, 1:10 and 1:20 (*w*/*w*). The Pearson correlation showed a very strong positive correlation (r = 0.9150, *** *p* < 0.001) between the activity of the Na^+^/K^+^-ATPase and the increasing concentration of the antivenom.

### 3.4. The Examination of PLA2 Activity in Vaa Venom and Neutralization by Antivenom

**Figure 11:**
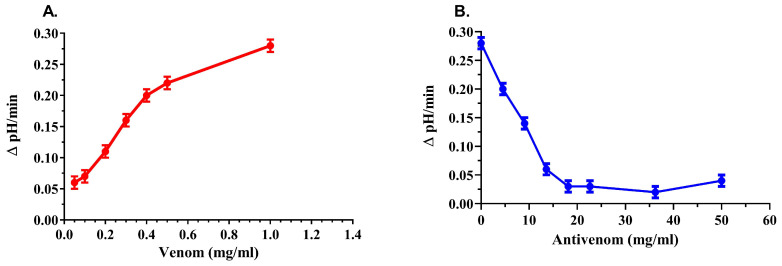
(**A**) Activity of the PLA2 in increasing concentrations of the *Vaa* venom (mg/mL); (**B**) Inhibition of the PLA2 activity in 1 mg/mL of the *Vaa* venom by increasing concentrations of the antivenom (mg/mL).

Under in vitro conditions, we investigated the activity of PLA2 in venom and the neutralization of its activity by antivenom based on the ΔpH over time using an egg yolk suspension as a substrate. The results are shown in Figure 11.

With an increase in the venom concentration in the substrate, there is an increase in ΔpH/min, which indicates an increase in PLA2 activity (Figure 11A). The addition of increasing concentrations of antivenom at a constant venom concentration (1 mg/mL) led to a decrease in the value of ΔpH/min (Figure 11B). This indicates a decrease in PLA2 activity, i.e., the neutralizing effect of the antivenom. Our results show that the activity of PLA2 in 1 mg of crude *Vaa* venom is completely neutralized by 18.12 mg of antivenom.

## 4. Discussion

Research into the composition of snake venom requires a multidisciplinary approach. Omics technologies, i.e., genomics, transcriptomics and proteomics, have recently played an increasingly important role in the field of venom research [43,44]. As a result of the application of these technologies, the number of new studies on the composition of snake venoms has increased significantly in recent years [45]. However, there is still a small number of proteomic studies on the venom of *V. ammodytes*. When reviewing the PubMed database (keywords: snake venom, proteomics, *V. ammodytes*), we found that a total of 10 papers were published for the period from 2008 to 2024. Various techniques for sample preparation and analysis were used in these studies, each with their own advantages and disadvantages. The analysis of the venom of Viperidae using the “*top-down*” technique leads to an incomplete characterization of the venom due to the presence of high-molecular-weight proteins that cannot be identified. In contrast, the “*bottom-up*” technique involves the prior trypsinization of the samples. “*Shotgun*” proteomics provides a quick qualitative overview of the composition of the venom but not the determination of its quantitative composition. This method often does not allow for the detection of numerous venom isoforms or the identification of small proteins and peptides [46]. Based on the above, it can be said that the limiting factors for the comparison of the venom compositions obtained in different studies are the following: the relatively small number of studies and the use of different proteomic techniques. In our study, we used the ‘bottom-up’ technique, which involves the trypsinization of the venom sample and the DiffPOP method to obtain the protein fractions of the venom (3A, 5A, 8A, 9A i 10A). In addition, data analysis against three databases (Serpentes, Vipera and Vipera ammodytes) and FDR < 1% provided identification of a higher number of proteins with improved confidence compared to previous studies of *Vaa* venom [47]. The results shown (Figure 2) clearly indicate an increase in the number of identified proteins when all fractions (3A, 5A, 8A, 9A and 10A) and the total venom (fraction 0) were analyzed, compared to the total venom (fraction 0). Since the UniProt database for *V. ammodytes* (Vipera ammodytes DB) is significantly smaller, as presented in the Section 2.2.4, we analyzed the mass spectrometry results using two additional databases: Vipera genus (Vipera DB) and Serpentes (Serpentes DB) (Figure 2A). As expected, the larger the DB used for searching, the higher the number of identified proteins, which we confirmed (Figure 2). Given that we use corrective measures (FDR < 1%), the number of false positive identifications has been reduced.

The composition of snake venoms in the family Viperidae can vary considerably between genera, species and even within a single species [48]. Snake venoms are mixtures of various proteins and non-protein components (such as amino acids, amines, nucleotides, metal ions, lipids and carbohydrates)—with or without toxic effects. Our results show that 97% of the dry mass of the *Vaa* venom consists of proteins, and proteomic analysis identified 159 different proteins originating from 26 protein families (Supplement S5, Table S10). It was interesting to compare our results with those of the proteomic analysis of *Vaa* venom from neighboring countries (Bulgaria, Croatia). In contrast to our results, the proteomic analysis of the venom *Vaa* from the area of Bulgaria identified 38 venom proteins originating from 9 protein families [49]. An analysis of the venom of *Vaa* from different parts of Croatia identified 57 proteins, which were classified into 16 protein families [50]. These data clearly show that there are differences in the composition of the *Vaa* venom even in geographically very close regions. In an earlier study on a proteomic analysis of the venom of *Vaa*, also from the territory of Serbia, it was reported that the number of identified proteins was 99 and the number of protein families was 9 [47].

Snake venom metalloproteinases (SvMPs) are largely responsible for the disruption of hemostasis [51]. In our study, 53 isoforms of this enzyme were detected in *Vaa* venom for the first time. Of these, 4 isoforms belonged to subclass PIII MPIII and 3 belonged isoforms to PII MPII, which is in agreement with the results of other authors [50,52,53]. Our results indicate the presence of a large amount of Zn^2+^-metalloproteinases disintegrin-like protein H4 subunit A (12 isoforms) and a large number of metalloproteinases (34 isoforms). Leonardi et al. [50] found that the relative abundance of SvMPS in the venom of *Vaa* from the area of Croatia is 20.40%, which is consistent with our results for the venom of *Vaa* from the area of Serbia. The SvSPs of *Vaa* venom exhibit high degrees of amino acid sequence similarity to the SvSPs of other Viperidae—for example, in *Macrovipera lebetina, Bitis gabonica, Daboia russelii* and *V. berus* [54,55]. In our proteome analysis, 22 different serine proteases were detected, which account for 1/10 of the total proteins in *Vaa* venom (Figure 3). The relative abundance of this enzyme in our study is consistent with the results of the venom of *Vaa* from the area of Croatia [50].

Snake C-type lectin-like proteins (Snaclecs) are non-enzymatic proteins that influence the following processes: thrombosis and hemostasis, cell adhesion, endocytosis and pathogen neutralization [56,57]. Our proteomic analysis has shown that Snaclecs are the most abundant non-enzymatic components in the venom of *Vaa* (Figure 3). In contrast, this protein was not detected in the venom of *Vaa* from Bulgaria [49], while it was detected in the venom of *Vaa* from Croatia by transcriptome analysis with a percentage of 13.8% [50], which is a slightly lower value compared to our results.

Disintegrins (DIS) are proteins found in the venoms of various *Vipera* species that selectively block the function of integrin receptors, which play an important role in tissue homeostasis [58]. Low-molecular, cysteine-rich disintegrins strongly inhibit platelet aggregation and thus prevent blood clotting [59]. The presence of this *Vaa* venom component in our study is higher than the results of Leonardi et al. [50]. According to other authors, DIS was not detected in the venom of *Vaa* from the territory of Serbia [47].

L-amino acid oxidases (LAAOs) lead to pathological effects that are primarily due to the release of hydrogen peroxide during LAAO activity [60]. Proteomic analysis have shown that the venom of *Vaa* contains 1.6% LAAO [50], which is more than 3.5 times lower than our results.

The relative abundance of CRISPs in *Vaa* venom is determined by our proteomic analysis, and the results are consistent with the findings of Leonardi et al. [50].

One of the potential hemotoxins found in a small percentage in the venom of *Vaa* is phosphodiesterase (PD). In addition, we have detected components that are less represented in the venom, such as venom nerve growth factors (VNGFs), coagulation factor X, cystatin, β-fibrinogenase, α-fibrinogenase, peroxiredoxin, hyaluronidase, aminopeptidase, renin, TATA box-binding protein, antihemoragic factor and natriuretic peptide. Some of them were not detected in earlier analyses of the venom of *Vaa* from Serbia, Croatia and Bulgaria [47,49,50].

Kunitz-type serine protease inhibitors (SPIs) have been identified in the venoms of Viperidae and Elapidae [29]. We detected four different Kunitz/BPTI peptides, one Kunitz/SPI and two MPI peptides. These peptides were not detected in the venom of the *Vaa* originating from Bulgaria [49].

PLA2, depending on the isoelectric point (pI), can be divided into acidic and basic isoforms, with the basic isoforms having a higher affinity for cell membranes and consequently a higher toxicity [61]. In our study, we have demonstrated the diversity of pI values of this enzyme by proteomic analysis. As a component of PLA2, amodytoxin A (AtxA) has the highest pI value compared to amoditoxin B and C and is also many times more toxic than the latter (Table 1). The overall abundance of PLA2 in the venom of the *Vaa* in our studies was lower compared to the venoms of the *Vaa* from Croatia and Bulgaria and amounted to 11.60%. Of these, 4.35% were neurotoxic amoditoxins (Atxs: A, B and C), while the remaining 7.25% were amoditins (Atns), which have no neurotoxic effect (Table 1). In this study, the relative abundance of amoditoxins and amoditins in the venom of *Vaa* is described in detail for the first time.

The most common neurotoxins are those that disrupt the somatic PNS by acting presynaptically (β-neurotoxins), postsynaptically (α-neurotoxins) or within the synaptic cleft [62,63]. In our working protocol, we used indirect and direct EFS to induce NPD contractions. Indirect EFS stimulates the presynaptic nerve terminal, resulting in the release of ACh, which activates the nicotinic acetylcholine receptor (nAChR) at the motor end-plate. This subsequently leads to the depolarization of the muscle cell and its contraction. Increasing concentrations of the competitive nAChR antagonist pancuronium (1 and 3 μM) reduce NPD contractions in a concentration-dependent manner, confirming with certainty that the parameters of indirect EFS are selective for the motor neuron. Direct EFS “bypasses” the neuromuscular synaptic cleft and triggers an NPD contraction through the direct activation of the contractile muscle machinery (actin–myosin interaction). Under the influence of *Vaa* venom, there was a progressive decrease in NPD contractions (Figure 5). The venom did not cause a change in the basal tension of the NPD. When we applied direct EFS parameters after an almost complete inhibition of indirect NPD contractions, the amplitude of NPD contractions reached values close to the control (Figure 5). This finding indicates that *Vaa* venom at a concentration that has a significant neurotoxic effect does not exhibit myotoxicity in the diaphragm and does not cause disturbances at the level of the contractile muscle machinery. This result is consistent with Logonder et al. [64], who investigated the neurotoxicity of ammodytoxin A from *Vaa* using an isolated mouse diaphragm model.

Figure 6 shows that the venom/antivenom mixture at the ratio of 1:2 reduces the neurotoxic effect of PLA2 but causes only a slight rightward shift of the time-dependent sigmoidal curve compared to the curve for the venom. The time-dependent sigmoidal curves for venom/antivenom mixtures at the ratios of 1:10 and 1:20 are clearly shifted to the right and have a lower slope than the curve for venom, especially the curve for the 1:20 mixture. In addition, there were highly significant (*p* < 0.001) differences between the ET_50_ values for all three venom/antivenom ratios (Figure 7). This proves that antivenom exerts a concentration-dependent protection of the decrease in NPD contractility as a function of time. Unfortunately, we were unable to compare this part of our results, as we found no studies in the literature that investigated the protective effect of the antivenom against the neurotoxic effects of the venom of *Vaa* using neuromuscular preparations of the diaphragm.

When examining NPD contractility, we noticed that the tetanic fade does not occur under the influence of *Vaa* venom (Figure 8C, white arrows). Presynaptic α3β2 nicotinic autoreceptors facilitate the release of ACh (Figure 8A, white arrows), ensuring reliable transmission at strategic sites such as neuromuscular synapses. Figure 8B (black arrows) shows that the non-depolarizing neuromuscular blocker pancuronium leads to the appearance of a tetanic fade NPD, which is a consequence of the blockade of presynaptic α3β2 autoreceptors. However, there is evidence that the occurrence of tetanic fade cannot be explained simply and only by the blockade of the α3β2 autoreceptors. Other receptors, such as muscarinic and purinergic receptors, coexist with this receptor at presynaptic nerve terminals and also play an important role in modulating ACh release [65,66,67]. For example, studies have demonstrated that α-conotoxin MII, a highly selective antagonist of α3β2, does not induce tetanic fade, although it leads to reduced ACh release [68]. This implies high safety margins in neuromuscular transmission. For the above reasons, we can only conclude that PLA2 did not lead to tetanic fade under our experimental conditions and that further investigation is required for additional interpretation.

There are two ways in which AChE activity can be significantly altered, leading to the disruption of physiological neurotransmission. The first possibility is that the venom has high AChE activity (exogenous AChE), which leads to the increased hydrolysis of ACh in the synaptic cleft, resulting in the inhibition of neurotransmission [13]. High AChE activity was found in the venoms of snakes from the Elapidae family. In contrast, no AChE activity was found in snakes from the families Viperidae and Crotalidae [69]. The second possibility is that the venom inhibits AChE in the synaptic cleft. For example, this is characteristic of venoms from the Elapidae family, which contain the neurotoxin fasciculin (genus *Dendroaspis*—mambas*, D. angusticeps*—green mamba, *D. polylepis*—black mamba, *D. viridis*—western green mamba, *D. jamesoni*—Jameson’s mamba). The inhibition of AChE leads to fasciculations and spasms of the striated muscles [9]. Based on our results, no statistically significant difference was found in AChE activity in the diaphragm under the influence of the venom and the venom/antivenom mixtures in all mass ratios compared to the control group (*p* > 0.05) (Figure 9). These results support the reports in the literature indicating that the enzyme AChE is not involved in the neurotoxic mechanism of action of the venom of Viperidae. This is also consistent with our proteomic analysis, which showed that AChE was not detected in *Vaa* venom.

The enzyme Na^+^/K^+^-ATPase (sodium-potassium pump) plays a decisive role in the generation of the concentration gradient across the cell membrane and the maintenance of the resting membrane potential. This enzyme consumes a considerable amount of energy for its function in many cells, especially in nerve and muscle cells. It is estimated that under standard resting conditions, 19 to 28% of total ATP in the body is used for Na^+^/K^+^-ATPase function. Under normal conditions, maintaining ionic gradients in brain neurons requires 50–60% of total oxygen consumption coupled to ATP synthesis, most of which is consumed by the Na^+^/K^+^-pump [70,71,72,73]. These data indicate that the lack of the cellular energy currency ATP leads to a significant reduction in the activity of Na+/K+-ATPase, particularly in nervous tissue. Ammodytoxin A (AtxA), the most toxic PLA2 of the *Vaa* venom, is selectively and rapidly internalized into the presynaptic terminals of motor neurons and then translocated into the mitochondrial intermembrane space and matrix [74,75]. Moreover, Ivanušec et al. [74] also found that the phospholipase activity of AtxA is not obligatory for its rapid intracellular and subcellular internalization. Since we have shown by the proteomic analysis of *Vaa* venom that AtxA is a protein with a low molecular weight of 15.5 kDa, we hypothesize that this could also be the reason for its rapid entry into mitochondrial structures. Mitochondria are very sensitive to fatty acids, lysophosphatides and PLA2, and their damage leads to a decrease in ATP production and the deenergization of terminal nerve endings [64]. As a result, the Na^+^/K^+^-ATPases lack energy support and their activity is inhibited. In this way, many important functions are disrupted, including the generation of axonal and synaptic membrane potentials and the mobilization of synaptic vesicles for the exocytosis of neurotransmitters [76]. In a study carried out on a neuromuscular preparation of the diaphragm, the neurotoxic effects of AtxA were investigated. Electron microscopy showed the most pronounced changes in the mitochondria of the presynaptic nerve endings. The mitochondria were swollen and damaged, with a disrupted mitochondrial cristae structure [64]. This finding supports our results showing that there is a highly significant (*p* < 0.001) inhibition of Na^+^/K^+^-ATPase in NPD under the influence of *Vaa* venom (Figure 10). The mixture with the highest proportion of antivenom (1:20) almost completely restored Na^+^/K^+^-ATPase activity (94.55% of the control). The Pearson correlation revealed a very strong positive correlation (r = 0.9150, *p* < 0.001) between the increasing activity of the Na^+^/K^+^-ATPase and the increasing concentration of the antivenom. Therefore, we conclude that the antivenom effectively prevents the Vaa venom from causing a reduction in Na+/K+-ATPase activity. We did not find any studies on the effect of Viperidae venom on Na^+^/K^+^-ATPase activity in NPD in the available literature.

We investigated the activity of PLA2 in *Vaa* venom under in vitro conditions, both in the absence and presence of the antivenom. The results show that the antivenom completely neutralizes the activity of PLA2 at a mass ratio of venom/antivenom of 1:18 (Figure 11B). We emphasize that this ratio corresponds approximately to the mass ratio of 1:20, which restores the activity of the Na^+^/K^+^-ATPase in the NPD to a level that is no longer statistically different from the control (Fugure 10). Due to the use of different methods to determine PLA2 enzyme activity, the use of different substrates and the expression of enzyme activity in different units, we were not able to compare the presented results with literature data.

## 5. Conclusions

With proteomic analysis, we have identified the complete composition of the *Vaa* venom, which gives us the opportunity to expand the database for *Vaa*. *Vaa* venom causes a progressive and significant decrease in the contractions of the NPD, without disturbance at the level of the contractile muscle machinery (actin–myosin interaction) and without the occurrence of a tetanic fade. The antivenom abolished the venom-induced progressive decrease in NPD contractions in a concentration-dependent manner by inhibiting PLA2 activity. Under the influence of *Vaa* venom, there is no change in AChE activity in the NPD, indicating that the neurotoxic mechanism of action of *Vaa* venom is not mediated by this enzyme. *Vaa* venom leads to a pronounced inhibition of Na^+^/K^+^-ATPase activity in the NPD. Antivenom with approximately the same mass proportion almost completely restores Na^+^/K^+^-ATPase activity in the NPD and completely neutralizes the PLA2 activity of the venom in vitro. Considering the phospholipase activity of PLA2, our further studies will focus on investigating the parameters of oxidative stress in NPD under the influence of this component and the potential protective effect of the antivenom.

## Figures and Tables

**Table 1:** Phospholipases (PLA2) of the venom of *V. ammodytes*.

Name	Mw (kDa)	pI	Relative Abundance of Protein Groups (%)	Enzyme Activity	Enzyme Action
Ammodytoxins—Atxs					Presynaptic neurotoxins
Ammodytoxin A (AtxA)	15.5	7.97	1.45	active	Most toxic
Ammodytoxin B (AtxB)	15.5	7.83	1.45	active	* 28 times less toxic than AtxA
Ammodytoxin C (AtxC)	15.5	7.62	1.45	active	* 17 times less toxic than AtxA
Ammodytins—Atns					
Ammodytin L (AtnL)	15.6	8.5	2.90	inactive	Myotoxic, cardiotoxic
Ammodytin I1 (AtnI1)	15.4	5.25	1.45	active	Non-toxic
Ammodytin I2 (AtnI2)	15.3 15.2	6.47 5.91	1.45 1.45	active	Non-toxic

* [42].

**Table 2:** Decrease in diaphragmatic contractility as a function of time under the influence of venom and venom/antivenom mixtures in different mass ratios (1:2; 1:10; 1:20).

Time (min)	The Mean Values of the NPD Contractions (%) Compared to the Control Contractions			
	Venom	Venom/Antivenom 1:2	Venom/Antivenom 1:10	Venom/Antivenom 1:20
15	90.95	96.55	97.78	93.60
30	79.13	84.61	89.51	89.92
45	64.15	72.20	76.26	86.08
60	52.68	61.47	71.13	88.27
75	40.74	46.64	66.01	81.50
90	31.49	40.67	63.21	80.76
105	24.35	34.79	60.30	74.09
120	16.56	30.50	54.86	72.49
135	14.93	21.64	48.91	68.71
150	12.30	18.00	36.56	67.11
165	10.36	13.15	29.45	67.06
180	8.78	10.54	22.61	67.26
195				61.65
210				58.79
225				56.90

**Table 3:** Calculated ET_50_ (minutes) after the administration of venom and venom/antivenom mixture at the ratios 1:2; 1:10 and 1:20 (*w*/*w*) (mean ± SD).

Venom and Venom/Antivenom Mixtures at Different Mass Ratios	Venom	Venom/Antivenom 1:2	Venom/Antivenom 1:10	Venom/Antivenom 1:20
ET_50_ (minutes)	60.17 ± 2.43	73.29 ± 3.02	114.80 ± 12.16	317.80 ± 40.46

## Data Availability

The original contributions presented in the study are included in the article/Supplementary Materials; further inquiries can be directed to the corresponding author/s.

## Supplementary Materials

The following supporting information can be downloaded at: https://www.mdpi.com/article/10.3390/vetsci11120605/s1, Supplement S1 (Table S1: Master proteins fractions 0); Supplement S2 (Table S2: Venom Vaa identified proteins Serpentes DB; Table S3: Venom Vaa identified proteins Vipera DB; Table S4: Venom Vaa identified proteins Vipera ammodytes DB); Supplement S3 (Table S5: Protein groups fraction 0 all DB; Table S6: Serpentes DB protein groups; Table S7: Vipera DB protein groups; Table S8: Vipera ammodytes DB protein groups); Supplement S4 (Table S9: Protein groups all DB); Supplement S5 (Table S10: Bioinformatics for a Total of 159 identified proteins); Supplement S6 (Figure S1: Total ion chromatograms (TIC) for the complete venom, fraction 0 (panel A) and for DiffPOP protein fractions 3A (panel B), 5A (panel C), 8A (panel D), 9A (panel E) and 10A (panel F); Figure S2: Representative recording of contractions of a neuromuscular preparation of the diaphragm (NPD) induced by indirect EFS and direct EFS under the influence of a mixture of venom and antivenom in a ratio of 1:2 (*w*/*w*); Figure S3: Representative recording of contractions of a neuromuscular preparation of the diaphragm (NPD) induced by indirect EFS and direct EFS under the influence of a mixture of venom and antivenom at a ratio of 1:10 (*w*/*w*); Figure S4: Representative recording of contractions of a neuromuscular preparation of the diaphragm (NPD) induced by indirect EFS and direct EFS under the influence of a mixture of venom and antivenom at a ratio of 1:20 (*w*/*w*)).

## References

[1] Minghui R., Malecela M.N., Cooke E., Abela-Ridder B. (2019). WHO’s Snakebite Envenoming Strategy for Prevention and Control. Lancet Glob. Health.

[2] Harrison R.A., Casewell N.R., Ainsworth S.A., Lalloo D.G. (2019). The Time Is Now: A Call for Action to Translate Recent Momentum on Tackling Tropical Snakebite into Sustained Benefit for Victims. Trans. R. Soc. Trop. Med. Hyg..

[3] Knudsen C., Ledsgaard L., Dehli R.I., Ahmadi S., Sørensen C.V., Laustsen A.H. (2019). Engineering and Design Considerations for Next-Generation Snakebite Antivenoms. Toxicon Off. J. Int. Soc. Toxinol..

[4] Tomović L., Anđelković M., Krizmanić I., Ajtić R., Urošević A., Labus N., Simović A., Maričić M., Golubović A., Ćorović J. (2019). Distribution of Three Vipera Species in the Republic of Serbia. Bull. Nat. Hist. Mus..

[5] Chan Y.S., Cheung R.C.F., Xia L., Wong J.H., Ng T.B., Chan W.Y. (2016). Snake Venom Toxins: Toxicity and Medicinal Applications. Appl. Microbiol. Biotechnol..

[6] Munawar A., Ali S.A., Akrem A., Betzel C. (2018). Snake Venom Peptides: Tools of Biodiscovery. Toxins.

[7] Pandit K., Rawal A., Maskey H.M.S., Nepal G. (2024). Neurological and Neuro-Ophthalmological Manifestations of Snake Bite: A Systematic Review. Ann. Med. Surg..

[8] Silva A., Hodgson W.C., Isbister G.K. (2017). Antivenom for Neuromuscular Paralysis Resulting From Snake Envenoming. Toxins.

[9] Ranawaka U.K., Lalloo D.G., de Silva H.J. (2013). Neurotoxicity in Snakebite--the Limits of Our Knowledge. PLoS Negl. Trop. Dis..

[10] Silva A., Maduwage K., Sedgwick M., Pilapitiya S., Weerawansa P., Dahanayaka N.J., Buckley N.A., Johnston C., Siribaddana S., Isbister G.K. (2016). Neuromuscular Effects of Common Krait (Bungarus Caeruleus) Envenoming in Sri Lanka. PLoS Negl. Trop. Dis..

[11] Silva A., Maduwage K., Sedgwick M., Pilapitiya S., Weerawansa P., Dahanayaka N.J., Buckley N.A., Siribaddana S., Isbister G.K. (2016). Neurotoxicity in Russell’s Viper (Daboia Russelii) Envenoming in Sri Lanka: A Clinical and Neurophysiological Study. Clin. Toxicol..

[12] Hodgson W.C., Wickramaratna J.C. (2002). *In Vitro* Neuromuscular Activity of Snake Venoms. Clin. Exp. Pharmacol. Physiol..

[13] Bickler P.E., Abouyannis M., Bhalla A., Lewin M.R. (2023). Neuromuscular Weakness and Paralysis Produced by Snakebite Envenoming: Mechanisms and Proposed Standards for Clinical Assessment. Toxins.

[14] Oliveira A.L., Viegas M.F., Da Silva S.L., Soares A.M., Ramos M.J., Fernandes P.A. (2022). The Chemistry of Snake Venom and Its Medicinal Potential. Nat. Rev. Chem..

[15] Castro-Amorim J., Novo de Oliveira A., Da Silva S.L., Soares A.M., Mukherjee A.K., Ramos M.J., Fernandes P.A. (2023). Na/K-ATPase_Catalytically Active Snake Venom PLA2 Enzymes: An Overview of Its Elusive Mechanisms of Reaction. J. Med. Chem..

[16] Bittenbinder M.A., van Thiel J., Cardoso F.C., Casewell N.R., Gutiérrez J.-M., Kool J., Vonk F.J. (2024). Tissue Damaging Toxins in Snake Venoms: Mechanisms of Action, Pathophysiology and Treatment Strategies. Commun. Biol..

[17] Tonello F., Simonato M., Aita A., Pizzo P., Fernández J., Lomonte B., Gutiérrez J.M., Montecucco C. (2012). A Lys49-PLA2 Myotoxin of Bothrops Asper Triggers a Rapid Death of Macrophages That Involves Autocrine Purinergic Receptor Signaling. Cell Death Dis..

[18] Šribar J., Oberčkal J., Križaj I. (2014). Understanding the Molecular Mechanism Underlying the Presynaptic Toxicity of Secreted Phospholipases A2: An Update. Toxicon.

[19] Kirakosyan G., Mohamadvarzi M., Ghulikyan L., Zaqaryan N., Kishmiryan A., Ayvazyan N. (2016). Morphological and Functional Alteration of Erythrocyte Ghosts and Giant Unilamellar Vesicles Caused by Vipera Latifi Venom. Comp. Biochem. Physiol. Part C Toxicol. Pharmacol..

[20] Jayaraman G., Krishnaswamy T., Kumar S., Yu C. (1999). Binding of Nucleotide Triphosphates to Cardiotoxin Analogue II from the Taiwan Cobra Venom (Naja Naja Atra). Elucidation of the Structural Interactions in the dATP-Cardiotoxin Analogue Ii Complex. J. Biol. Chem..

[21] Kumar T.K.S., Jayaraman G., Lee C.S., Arunkumar A.I., Sivaraman T., Samuel D., Yu C. (1997). Snake Venom Cardiotoxins-Structure, Dynamics, Function and Folding. J. Biomol. Struct. Dyn..

[22] Bougis P.E., KhElif A., Rochat H. (1989). On the Inhibition of [Na+,K+]-ATPasesby the Components of Naja Mossambica Activities Mossambica Venom: Evidence for Two Distinct Rat Brain [Na+,K+]-ATPase. Biochemistry.

[23] Kaplia A.A., Kravtsova V.V., Kravtsov A.V. (1996). [Effect of phospholipase A2 from Naja naja oxiana venom on activity of Na+,K+-ATPase isoenzymes in rat brain]. Biokhimiia Mosc. Russ..

[24] Leite R.S., Pinheiro G.H.D., Fernandes M.N., Selistre-de-Araujo H.S. (2006). The Effect of the Myotoxic Lys49 Phospholipase A(2) from Agkistrodon Contortrix Laticinctus Snake Venom on Na+/K+ -ATPase Activity of Toad Bladders. Toxicol. Vitro Int. J. Publ. Assoc. BIBRA.

[25] Leite R.S., Franco W., Ownby C.L., Selistre-de-Araujo H.S. (2004). Effects of ACL Myotoxin, a Lys49 Phospholipase A(2) from Agkistrodon Contortrix Laticinctus Snake Venom, on Water Transport in the Isolated Toad Urinary Bladder. Toxicon Off. J. Int. Soc. Toxinol..

[26] Lamb T., De Haro L., Lonati D., Brvar M., Eddleston M. (2017). Antivenom for European *Vipera* Species Envenoming. Clin. Toxicol..

[27] Dobaja Borak M., Babić Ž., Caganova B., Grenc D., Karabuva S., Kolpach Z., Krakowiak A., Kolesnikova V., Lukšić B., Pap C. (2023). Viper Envenomation in Central and Southeastern Europe: A Multicentre Study. Clin. Toxicol..

[28] Latinović Z., Leonardi A., Šribar J., Sajevic T., Žužek M.C., Frangež R., Halassy B., Trampuš-Bakija A., Pungerčar J., Križaj I. (2016). Venomics of Vipera Berus Berus to Explain Differences in Pathology Elicited by Vipera Ammodytes Ammodytes Envenomation: Therapeutic Implications. J. Proteom..

[29] Giribaldi J., Kazandjian T., Amorim F.G., Whiteley G., Wagstaff S.C., Cazals G., Enjalbal C., Quinton L., Casewell N.R., Dutertre S. (2020). Venomics of the Asp Viper Vipera Aspis Aspis from France. J. Proteom..

[30] Georgieva D., Arni R.K., Betzel C. (2008). Proteome Analysis of Snake Venom Toxins: Pharmacological Insights. Expert Rev. Proteom..

[31] Tasoulis T., Isbister G.K. (2023). A Current Perspective on Snake Venom Composition and Constituent Protein Families. Arch. Toxicol..

[32] Lukic I., Blagojevic V., Minic R., Ivanovic S., Borozan S., Cupic V., Zivkovic I. (2024). Comparison of Cytotoxicity Methods for Studying Vipera Ammodytes Venom and the Anticytotoxic Potency of Antivenom. Cent.-Eur. J. Immunol..

[33] Pinto A.F.M., Diedrich J.K., Moresco J.J., Yates J.R. (2023). Differential Precipitation of Proteins: A Simple Protein Fractionation Strategy to Gain Biological Insights with Proteomics. J. Am. Soc. Mass Spectrom..

[34] Rešetar Maslov D., Rubić I., Farkaš V., Kuleš J., Beer Ljubić B., Beletić A., Samardžija M., Kovačić M., Jurkić Krsteska G., Mrljak V. (2024). Characterization and LC-MS/MS Based Proteomic Analysis of Extracellular Vesicles Separated from Blood Serum of Healthy and Dogs Naturally Infected by Babesia Canis. A Preliminary Study. Vet. Parasitol..

[35] Rešetar Maslov D., Farkaš V., Rubić I., Kuleš J., Beletić A., Beer Ljubić B., Šmit I., Mrljak V., Torti M. (2023). Serum Proteomic Profiles Reflect the Stages of Myxomatous Mitral Valve Disease in Dogs. Int. J. Mol. Sci..

[36] Gutiérrez J.M., Solano G., Pla D., Herrera M., Segura Á., Vargas M., Villalta M., Sánchez A., Sanz L., Lomonte B. (2017). Preclinical Evaluation of the Efficacy of Antivenoms for Snakebite Envenoming: State-of-the-Art and Challenges Ahead. Toxins.

[37] Krummer S., Thiermann H., Worek F., Eyer P. (2002). Equipotent Cholinesterase Reactivation in Vitro by the Nerve Agent Antidotes HI 6 Dichloride and HI 6 Dimethanesulfonate. Arch. Toxicol..

[38] Ellman G.L., Courtney K.D., Andres V., Feather-Stone R.M. (1961). A New and Rapid Colorimetric Determination of Acetylcholinesterase Activity. Biochem. Pharmacol..

[39] Lowry O.H., Rosebrough N.J., Farr A.L., Randall R.J. (1951). Protein Measurement with the Folin Phenol Reagent. J. Biol. Chem..

[40] Pari L., Murugavel P. (2007). Diallyl Tetrasulfide Improves Cadmium Induced Alterations of Acetylcholinesterase, ATPases and Oxidative Stress in Brain of Rats. Toxicology.

[41] Tan N.H., Tan C.S. (1988). Acidimetric Assay for Phospholipase A Using Egg Yolk Suspension as Substrate. Anal. Biochem..

[42] Prijatelj P., Copic A., Krizaj I., Gubensek F., Pungercar J. (2000). Charge Reversal of Ammodytoxin A, a Phospholipase A2-Toxin, Does Not Abolish Its Neurotoxicity. Biochem. J..

[43] Kerkkamp H.M.I., Kini R.M., Pospelov A.S., Vonk F.J., Henkel C.V., Richardson M.K. (2016). Snake Genome Sequencing: Results and Future Prospects. Toxins.

[44] Modahl C.M., Brahma R.K., Koh C.Y., Shioi N., Kini R.M. (2020). Omics Technologies for Profiling Toxin Diversity and Evolution in Snake Venom: Impacts on the Discovery of Therapeutic and Diagnostic Agents. Annu. Rev. Anim. Biosci..

[45] Damm M., Hempel B.-F., Süssmuth R.D. (2021). Old World Vipers-A Review about Snake Venom Proteomics of Viperinae and Their Variations. Toxins.

[46] Petras D., Hempel B.-F., Göçmen B., Karis M., Whiteley G., Wagstaff S.C., Heiss P., Casewell N.R., Nalbantsoy A., Süssmuth R.D. (2019). Intact Protein Mass Spectrometry Reveals Intraspecies Variations in Venom Composition of a Local Population of Vipera Kaznakovi in Northeastern Turkey. J. Proteom..

[47] Gopcevic K., Karadzic I., Izrael-Zivkovic L., Medic A., Isakovic A., Popović M., Kekic D., Stanojkovic T., Hozic A., Cindric M. (2021). Study of the Venom Proteome of Vipera Ammodytes Ammodytes (Linnaeus, 1758): A Qualitative Overview, Biochemical and Biological Profiling. Comp. Biochem. Physiol. Part D Genom. Proteom..

[48] Casewell N.R., Jackson T.N.W., Laustsen A.H., Sunagar K. (2020). Causes and Consequences of Snake Venom Variation. Trends Pharmacol. Sci..

[49] Georgieva D., Risch M., Kardas A., Buck F., von Bergen M., Betzel C. (2008). Comparative Analysis of the Venom Proteomes of Vipera Ammodytes Ammodytes and Vipera Ammodytes Meridionalis. J. Proteome Res..

[50] Leonardi A., Sajevic T., Pungerčar J., Križaj I. (2019). Comprehensive Study of the Proteome and Transcriptome of the Venom of the Most Venomous European Viper: Discovery of a New Subclass of Ancestral Snake Venom Metalloproteinase Precursor-Derived Proteins. J. Proteome Res..

[51] Sajevic T., Leonardi A., Križaj I. (2014). An Overview of Hemostatically Active Components of *Vipera Ammodytes Ammodytes* Venom. Toxin Rev..

[52] Sanchez E.F., Flores-Ortiz R.J., Alvarenga V.G., Eble J.A. (2017). Direct Fibrinolytic Snake Venom Metalloproteinases Affecting Hemostasis: Structural, Biochemical Features and Therapeutic Potential. Toxins.

[53] Gutiérrez J.M., Escalante T., Rucavado A., Herrera C. (2016). Hemorrhage Caused by Snake Venom Metalloproteinases: A Journey of Discovery and Understanding. Toxins.

[54] Siigur E., Aaspõllu A., Siigur J. (2001). Sequence Diversity of Vipera Lebetina Snake Venom Gland Serine Proteinase Homologs--Result of Alternative-Splicing or Genome Alteration. Gene.

[55] Calvete J.J., Marcinkiewicz C., Sanz L. (2007). Snake Venomics of Bitis Gabonica Gabonica. Protein Family Composition, Subunit Organization of Venom Toxins, and Characterization of Dimeric Disintegrins Bitisgabonin-1 and Bitisgabonin-2. J. Proteome Res..

[56] Ogawa T., Chijiwa T., Oda-Ueda N., Ohno M. (2005). Molecular Diversity and Accelerated Evolution of C-Type Lectin-like Proteins from Snake Venom. Toxicon Off. J. Int. Soc. Toxinol..

[57] van den Berg L.M., Gringhuis S.I., Geijtenbeek T.B.H. (2012). An Evolutionary Perspective on C-Type Lectins in Infection and Immunity. Ann. N. Y. Acad. Sci..

[58] Calvete J.J., Marcinkiewicz C., Monleón D., Esteve V., Celda B., Juárez P., Sanz L. (2005). Snake Venom Disintegrins: Evolution of Structure and Function. Toxicon Off. J. Int. Soc. Toxinol..

[59] Calvete J.J. (2013). The Continuing Saga of Snake Venom Disintegrins. Toxicon Off. J. Int. Soc. Toxinol..

[60] Milovanovic V., Minic R., Vakic J., Ivanovic S., Cupic V., Borozan S., Nesic A., Zivkovic I. (2021). MTT Based L-Aminoacid Oxidase Activity Test for Determination of Antivenom Potency against Vipera Ammodytes Envenomation. Toxicon Off. J. Int. Soc. Toxinol..

[61] Gutiérrez J.M., Lomonte B. (2013). Phospholipases A2: Unveiling the Secrets of a Functionally Versatile Group of Snake Venom Toxins. Toxicon Off. J. Int. Soc. Toxinol..

[62] Osipov A., Utkin Y. (2012). Effects of Snake Venom Polypeptides on Central Nervous System. Cent. Nerv. Syst. Agents Med. Chem..

[63] AlShammari A.K., Abd El-Aziz T.M., Al-Sabi A. (2023). Snake Venom: A Promising Source of Neurotoxins Targeting Voltage-Gated Potassium Channels. Toxins.

[64] Logonder U., Krizaj I., Rowan E.G., Harris J.B. (2008). Neurotoxicity of Ammodytoxin a in the Envenoming Bites of Vipera Ammodytes Ammodytes. J. Neuropathol. Exp. Neurol..

[65] Lu Z., Yu B. (2007). Role of Presynaptic Acetylcholine Autoreceptors at Motor Nerve Endings on Tetanic and Train-of-Four Fade Seen during a Nondepolarizing Neuromuscular Block. Anesthesiology.

[66] Jonsson M., Eriksson L.I. (2007). Role of Presynaptic Acetylcholine Autoreceptors at Motor Nerve Endings on Tetanic and Train-of-Four Fade Seen during a Nondepolarizing Neuromuscular Block. Anesthesiology.

[67] Alves-do-Prado W., Corrado A.P., Prado W.A. (1987). Reversal by Atropine of Tetanic Fade Induced in Cats by Antinicotinic and Anticholinesterase Agents. Anesth. Analg..

[68] Faria M., Oliveira L., Timóteo M.A., Lobo M.G., Correia-De-Sá P. (2003). Blockade of Neuronal Facilitatory Nicotinic Receptors Containing Alpha 3 Beta 2 Subunits Contribute to Tetanic Fade in the Rat Isolated Diaphragm. Synapse.

[69] Frobert Y., Créminon C., Cousin X., Rémy M.H., Chatel J.M., Bon S., Bon C., Grassi J. (1997). Acetylcholinesterases from Elapidae Snake Venoms: Biochemical, Immunological and Enzymatic Characterization. Biochim. Biophys. Acta.

[70] Benziane B., Björnholm M., Pirkmajer S., Austin R.L., Kotova O., Viollet B., Zierath J.R., Chibalin A.V. (2012). Activation of AMP-Activated Protein Kinase Stimulates Na+,K+-ATPase Activity in Skeletal Muscle Cells. J. Biol. Chem..

[71] Rolfe D.F., Brown G.C. (1997). Cellular Energy Utilization and Molecular Origin of Standard Metabolic Rate in Mammals. Physiol. Rev..

[72] Erecińska M., Silver I.A. (1989). ATP and Brain Function. J. Cereb. Blood Flow Metab. Off. J. Int. Soc. Cereb. Blood Flow Metab..

[73] Clausen T., Van Hardeveld C., Everts M.E. (1991). Significance of Cation Transport in Control of Energy Metabolism and Thermogenesis. Physiol. Rev..

[74] Ivanušec A., Šribar J., Veranič P., Križaj I. (2022). The Phospholipase Activity of Ammodytoxin, a Prototype Snake Venom β-Neurotoxin, Is Not Obligatory for Cell Internalisation and Translocation to Mitochondria. Toxins.

[75] Logonder U., Jenko-Praznikar Z., Scott-Davey T., Pungercar J., Krizaj I., Harris J.B. (2009). Ultrastructural Evidence for the Uptake of a Neurotoxic Snake Venom Phospholipase A2 into Mammalian Motor Nerve Terminals. Exp. Neurol..

[76] Sheng Z.-H., Cai Q. (2012). Mitochondrial Transport in Neurons: Impact on Synaptic Homeostasis and Neurodegeneration. Nat. Rev. Neurosci..

