# Supplementary material for: The Venom of Vipera ammodytes ammodytes: Proteomics, Neurotoxic Effect and Neutralization by Antivenom
Source: Vet Sci. 2024 Nov 28;11(12):605. doi: 10.3390/vetsci11120605 (PMC11680118; doi:10.3390/vetsci11120605)
Supplement: Supplementary file 1 [file vetsci-11-00605-s001.zip › Titles of Supplements.pdf]

Supplement 1 (Table_S1_Master_proteins_fractions_0)
Supplement 2 (Table_S2_Venom_Vaa_identified_proteins_Serpentes_DB; Table_S3_Venom_Vaa_identified_proteins_Vipera_DB; Table_S4_Venom_Vaa_identified_proteins_Vipera_ammodytes_DB)
Supplement 3 (Table_S5_Protein_groups_fraction_0_all_DB; Table_S6_Serpentes_DB_protein_groups; Table_S7_Vipera_DB_protein_groups; Table_S8_Vipera_ammodytes_DB_protein_groups)
Supplement 4 (Table_S9_Protein_groups_all_DB)
Supplement 5 (Table_S10_Bioinformatics_for_a_Total_of_159_identified_proteins)

Figure_S_1. Total ion chromatograms (TIC) for the complete venom, fraction 0 (panel A) and for DiffPOP protein fractions 3A (panel B), 5A (panel C), 8A (panel D), 9A (panel E), and 10A (panel F).

Figure_S_2. Representative recording of contractions of a neuromuscular preparation of the diaphragm (NPD) induced by indirect EFS (·····) and direct EFS (**-----**) under the influence of a mixture of venom and antivenom in a ratio of 1:2 (w/w)

C_1 – C_5 - control contractions; 12 "packages" of contractions induced by indirect EFS; 2 "packages" of contractions induced by direct EFS

Figure_S_3. Representative recording of contractions of a neuromuscular preparation of the diaphragm (NPD) induced by indirect EFS (·····) and direct EFS (**-----**) under the influence of a mixture of venom and antivenom in a ratio of 1:10 (w/w)

C_1 – C_5 - control contractions; 12 "packages" of contractions induced by indirect EFS; 2 "packages" of contractions induced by direct EFS

Figure_S_4. Representative recording of contractions of a neuromuscular preparation of the diaphragm (NPD) induced by indirect EFS (·····) and direct EFS (**-----**) under the influence of a mixture of venom and antivenom in a ratio of 1:20 (w/w)

C_1 – C_5 - control contractions; 12 "packages" of contractions induced by indirect EFS; 2 "packages" of contractions induced by direct EFS
