# Supplementary figures and images for: The Venom of Vipera ammodytes ammodytes: Proteomics, Neurotoxic Effect and Neutralization by Antivenom
Source: Vet Sci. 2024 Nov 28;11(12):605. doi: 10.3390/vetsci11120605 (PMC11680118; doi:10.3390/vetsci11120605)

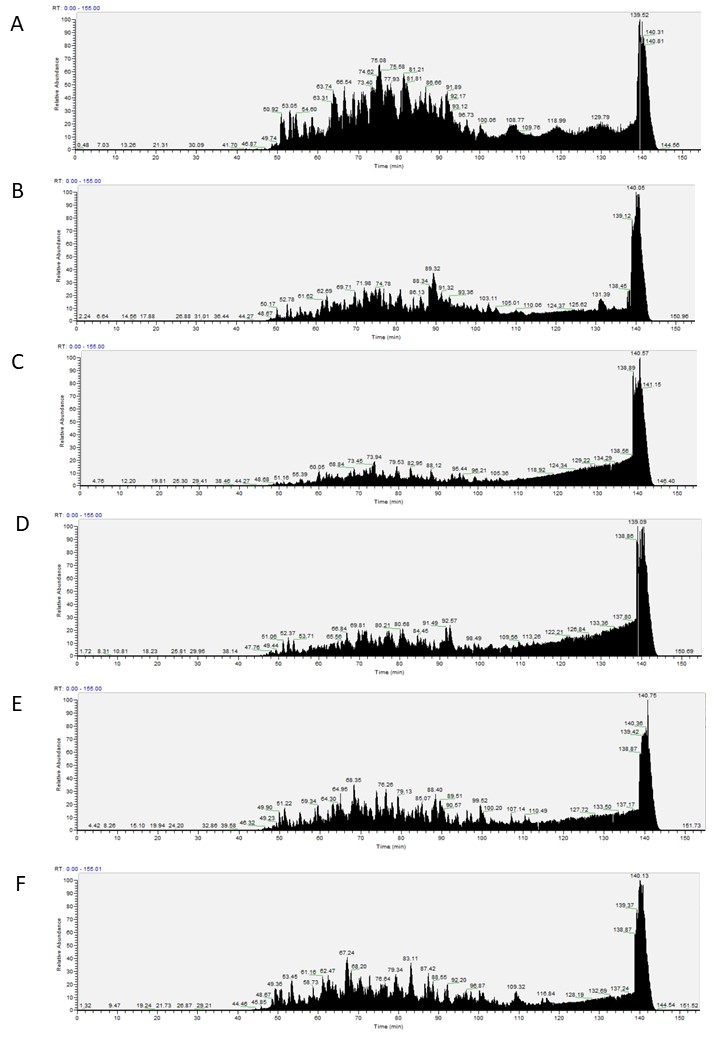

Supplement: Supplementary file 1 [file vetsci-11-00605-s001.zip › Figure_S1_TIC_.jpg]

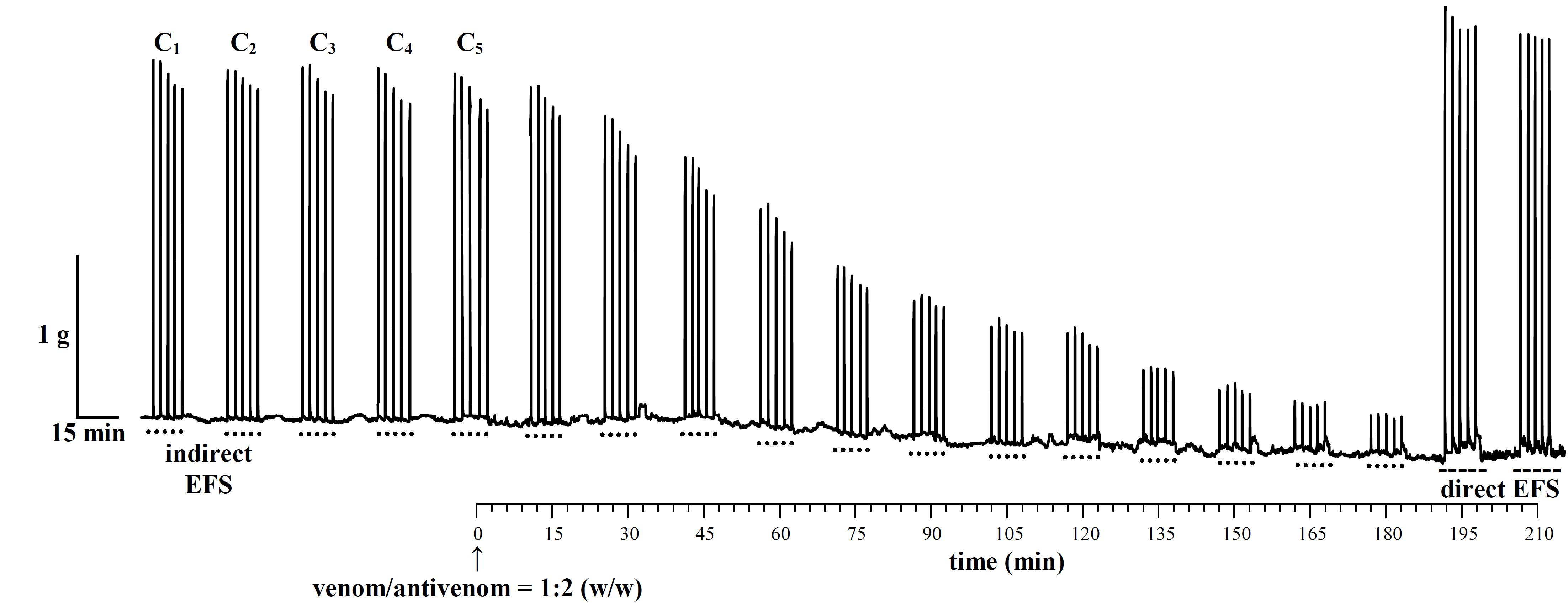

Supplement: Supplementary file 1 [file vetsci-11-00605-s001.zip › Figure_S2_NPD contractions under the influence of venom+antivenom=1_2.tif]

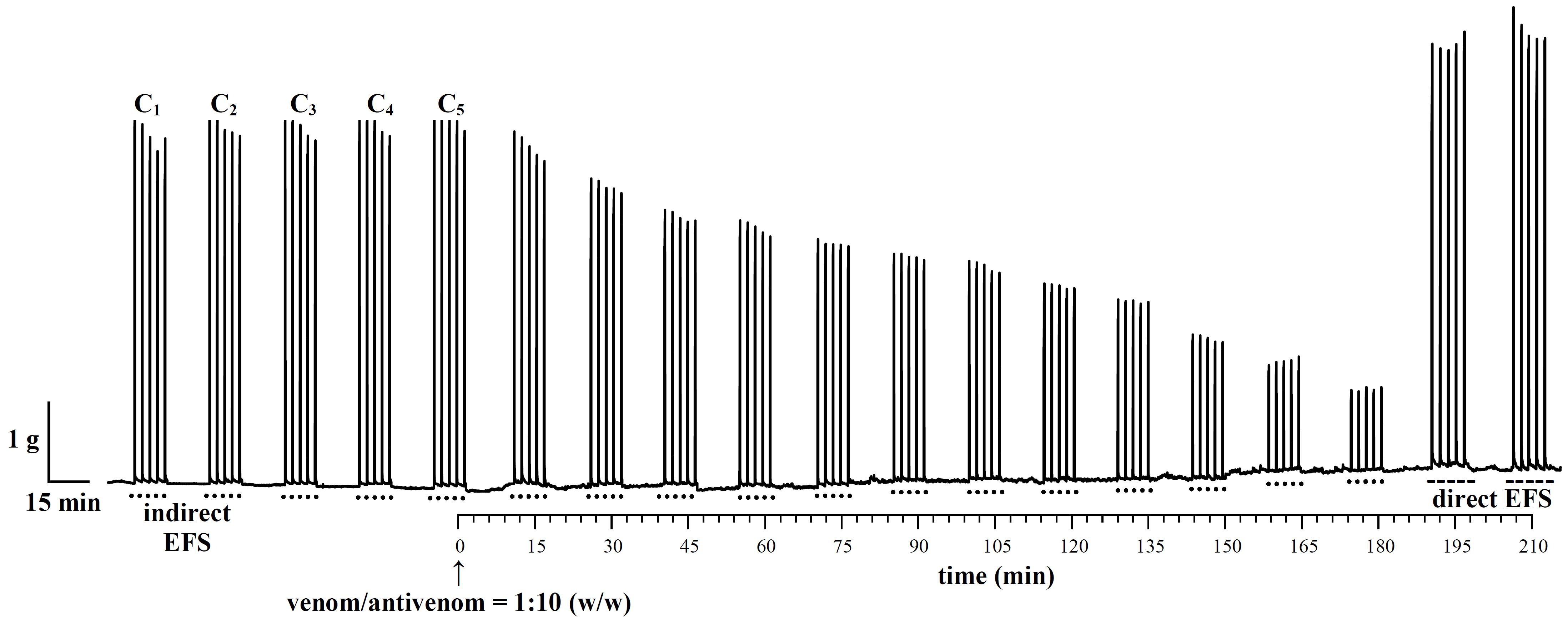

Supplement: Supplementary file 1 [file vetsci-11-00605-s001.zip › Figure_S3_NPD contractions under the influence of venom+antivenom=1_10.tif]

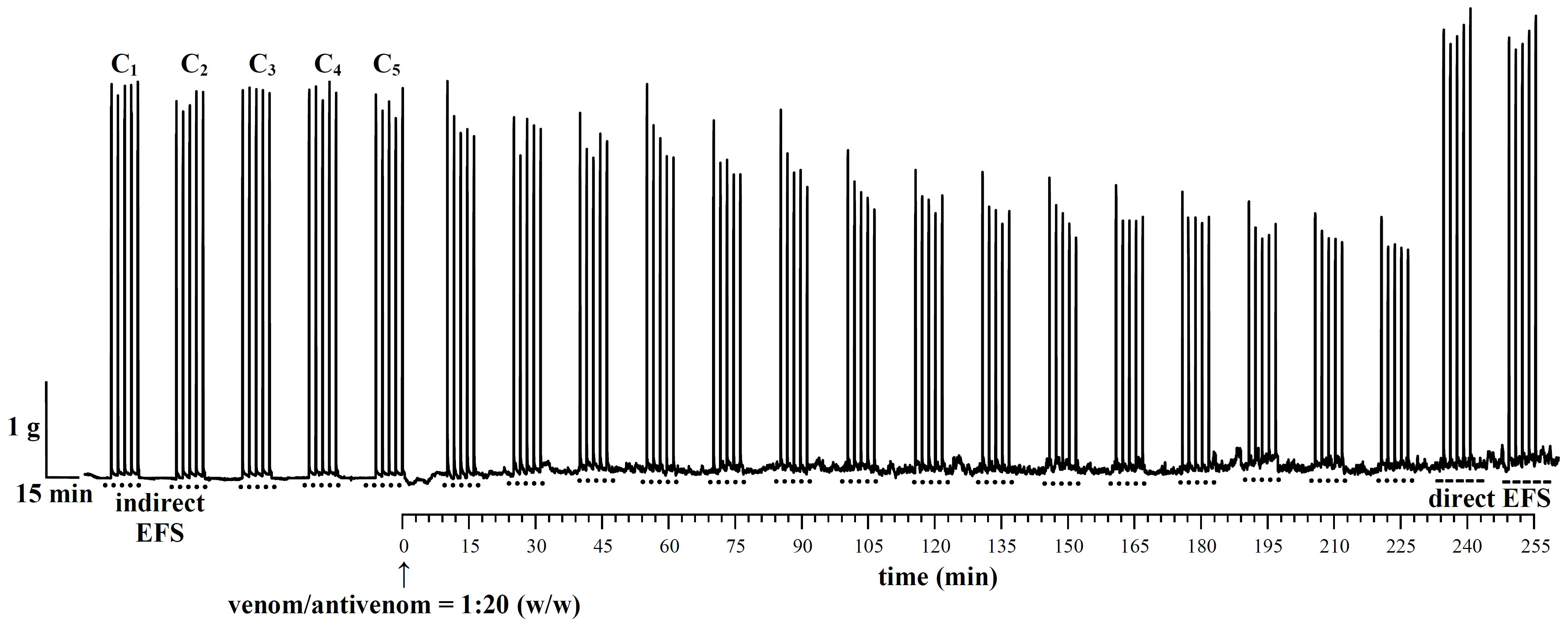

Supplement: Supplementary file 1 [file vetsci-11-00605-s001.zip › Figure_S4_NPD contractions under the influence of a mixture of venom and antivenom in a ratio of 1_20 (ww).tif]
